# Antibiotics: An overview on the environmental occurrence, toxicity, degradation, and removal methods

**DOI:** 10.1080/21655979.2021.1974657

**Published:** 2021-10-06

**Authors:** Qiulian Yang, Yuan Gao, Jian Ke, Pau Loke Show, Yuhui Ge, Yanhua Liu, Ruixin Guo, Jianqiu Chen

**Affiliations:** a School of Engineering, China Pharmaceutical University, Nanjing, 211198, China; b Department of Chemical and Environmental Engineering, Faculty of Science and Engineering, University of Nottingham Malaysia, Jalan Broga, Semenyih, Selangor Darul Ehsan, 43500, Malaysia

**Keywords:** Antibiotics, environmental occurrence, toxicity assessment, removal method, biodegradation

## Abstract

Antibiotics, as antimicrobial drugs, have been widely applied as human and veterinary medicines. Recently, many antibiotics have been detected in the environments due to their mass production, widespread use, but a lack of adequate treatment processes. The environmental occurrence of antibiotics has received worldwide attention due to their potential harm to the ecosystem and human health. Research status of antibiotics in the environment field is presented by bibliometrics. Herein, we provided a comprehensive overview on the following important issues: (1) occurrence of antibiotics in different environmental compartments, such as wastewater, surface water, and soil; (2) toxicity of antibiotics toward non-target organisms, including aquatic and terrestrial organisms; (3) current treatment technologies for the degradation and removal of antibiotics, including adsorption, hydrolysis, photodegradation and oxidation, and biodegradation. It was found that macrolides, fluoroquinolones, tetracyclines, and sulfonamides were most frequently detected in the environment. Compared to surface and groundwaters, wastewater contained a high concentration of antibiotic residues. Both antibiotics and their metabolites exhibited toxicity to non-target organisms, especially aquatic organisms (e.g., algae and fish). Fluoroquinolones, tetracyclines, and sulfonamides can be removed through abiotic process, such as adsorption, photodegradation, and oxidation. Fluoroquinolones and sulfonamides can directly undergo biodegradation. Further studies on the chronic effects of antibiotics at environmentally relevant concentrations on the ecosystem were urgently needed to fully understand the hazards of antibiotics and help the government to establish the permissible limits. Biodegradation is a promising technology; it has numerous advantages such as cost-effectiveness and environmental friendliness.

## 1. Introduction

Antibiotics are among the most commonly used drugs worldwide, which are capable of killing or inhibiting the growth of bacteria to fight bacterial infections. The emergence of antibiotics in the environment has attracted considerable attention in recent years due to their potential harm to humans and ecosystems [1]. Antibiotics are chemical substances derived from natural, semi-synthetic and synthetic sources. The first antibiotics are natural compounds produced by microorganism, e.g., penicillin extracted from the *Penicillium notatum* culture [2]. There are three ways to classify antibiotics. Firstly, antibiotics can be categorized into different classes based on their chemical structure, such as β-lactams, macrolides (MAs), fluoroquinolones (FQs), tetracyclines (TCs), and sulfonamides (SAs). Additionally, antibiotics can be divided into three classes according to their spectrum of activity, i.e., narrow-spectrum antibiotic, broad-spectrum antibiotic, and extended-spectrum antibiotic. Antibiotics can also be categorized by their mechanisms of action, involving bactericidal action (killing bacteria) and bacteriostatic action (inhibiting the growth of bacterial cells). For instance, β-lactam antibiotics (including penicillins and cephalosporins) can kill the bacteria by inhibiting the synthesis of bacterial cell walls; MAs and TCs have broad-spectrum activities and inhibit the bacterial growth through the inhibition of bacterial protein synthesis; FQs are synthetic broad-spectrum antibiotics and kill the bacteria via the interference with bacterial DNA synthesis; SAs are synthetic broad-spectrum bacteriostatic antibiotics and produce their effects by inhibiting the production of tetrahydrofolic acid required for nucleic acid synthesis. Commonly, SAs are used in combination with trimethoprim (TMP) which is a sulfonamide synergist and inhibits the enzyme dihydrofolate reductase [2–4]. So far, more than 250 antibiotics have been registered and used; commonly used antibiotics in human and veterinary medicines include the classes of β-lactams, MAs, FQs, TCs, and SAs [2,5,6].

Antibiotics have been extensively used for the treatment and prevention of diseases in humans, as well as for protecting animal health and promoting animal growth in aquaculture and livestock industries [2,7]. Recently, due to the global growth in human population and rising demand for animal protein [2], antibiotic consumption has been growing and reached enormous amounts. The use of antibiotics by humans showed an increase of 65% from 2000 to 2015, the consumption was predicted to increase to 200% by 2030 [1,8]. Previous research showed that it was estimated global consumption of 63,151 tons of antibiotics in livestock in 2010. Moreover, the proportion of veterinary antibiotics was predicted to increase by 67% (up to 105,596 tons) in 2030 due to growing consumer demand for livestock products [9]. In China itself, as the largest producer, exporter and user of antibiotics in the world, the antibiotic consumption is in large quantities due to the high populations and upward demand for animal protein. In 2013, total usage for 36 selected antibiotics in China was 92,700 tons and the number of antibiotics excreted by humans and animals was an estimated 54,000 tons, with 53,800 tons discharged into the environment [10].

Clearly, the widespread consumption of antibiotics has been accompanied by their continuous excretion and release into the environment. Antibiotics cannot be completely metabolized by humans and animals, nor can they be fully removed by wastewater treatment plants (WWTPs) [11,12]. As a result, both the unchanged parent compounds and their metabolites enter the environment. For example, TCs are not easily metabolized by humans and animals, such that >70% of the parent compound is excreted and released into the environment [13]. Similarly, up to 70% of unmetabolized FQs are excreted via the urine and feces, and enter the environment [14]. Considering the low concentrations of antibiotics present in the environment and the complication of environmental sample matrices, antibiotic extraction is a very important step prior to analysis. A variety of extraction techniques have been applied, such as solid-phase extraction (SPE) [15], ultrasound-assisted extraction (UAE) [16], QuEChERS extraction [17], and liquid–liquid microextraction [18]. The SPE and the UAE coupled with the SPE are frequently used to extract antibiotics from aqueous environmental matrices and solid environmental matrices, respectively, due to their easy operation and high efficiency [15–17,19,20]. Two main parameters (sorbent type and elution solvent) are critical in ensuring the effectiveness of SPE. A copolymer of divinylbenzene and vinylpyrrolidone (which can be obtained from waters under the trade name of Oasis HLB) is one of the most popular sorbents; the commonly used elution solvents are methanol and acetonitrile [5,15,16]. In addition, analytical methods based on liquid chromatography (LC) have been applied to selectively and sensitively detect the antibiotics present in the environment. Currently, due to the high sensitivity of tandem mass spectrometry (MS/MS) detection (detection limit: ng/L), LC-MS/MS detection are widely employed in the analysis of antibiotics in different environmental matrices after the samples clean-up and preconcentration performed by SPE. For example, the SPE-LC-MS/MS method was applied to analyze six antibiotics [amoxicillin (AMX), ciprofloxacin (CIP), trimethoprim (TMP), norfloxacin (NOR), sulfamethoxazole (SMX), and doxycycline (DOX)] in the wastewaters, surface waters and sediments, and achieved very low limit of quantification (LOQ) [7 ng/L (TMP) – 135 ng/L (DOX)] [15]. Similarly, the determinations of five antibiotic classes [MAs, FQs, TCs, SAs and chloramphenicols (CAPs)] in the surface water and sediments were performed by LC-MS/MS method and it was found that the LOQs were in the range of 0.03–1.68 g/L and 0.01–0.56 ng/g for water samples and sediment samples, respectively [21].

Recently, the detection of antibiotics present in different environmental matrices was achieved through chemical analysis based on the reliable and sensible instrumental methods [5]. Although the environmental concentrations of antibiotics are often at trace levels [ng/L (ng/g) – μg/L (μg/g)] [2,22], the emergence of antibiotics may pose a risk to non-target aquatic organisms and terrestrial plants. Protection of the living organisms in an ecosystem is of great importance in sustainable development of ecology and society. Considering the sustainable development goals (SDGs) 2030 Agenda (e.g., SDG 14 on life below water, and SDG 15 on life on land), our study summarized the occurrence of antibiotics in aquatic and terrestrial environments, and toxic effects of antibiotics toward the non-target organisms. We hope that this review can help the general public fully understand the hazards of antibiotics present in the environment and provide some references to the government to set the permissible limits of antibiotic discharge. Additionally, the discharge of antibiotic wastewater contributes significantly to water pollution and antibiotic resistance genes (ARGs) dissemination, and further impacts the accomplishment of the SDGs (e.g., SDG 3 on good health and well-being, and SDG 6 on clean water and sanitation). To control and mitigate antibiotic pollution, the recent advances on the degradation and removal methods of antibiotics were also presented in our review.

**Figure 1.**
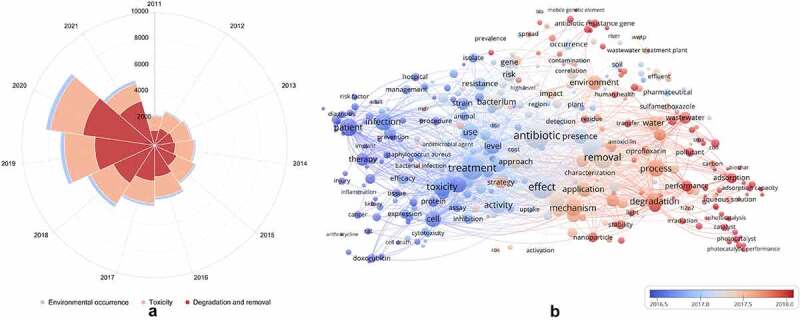
Annual number of publications (a) and cooccurrence network map of keywords (b) about researches on the environmental occurrence, toxicity, degradation, and removal of antibiotics during the last 10 years (from 2011 to 2021)

In addition, a bibliometric overview of studies on the environmental occurrence, toxicity, degradation, and removal of antibiotics is shown in Figure 1, and covers the studies performed from 2011 to 11 August 2021. The data are collected from the ‘All databases’ in the Web of Science, Clarivate Analytics, and the total number of publications was 49,216. As shown in Figure 1a, the total number of publications related to these three research directions increased yearly, from 2,305 publications in 2011 to 8,276 publications in 2020. Furthermore, compared to the publications in 2011, the number of articles in 2020 related to the environmental occurrence, toxicity, degradation and removal methods significantly increased by 2.90, 1.26, and 3.86 times, respectively. These results indicated that these three research directions have attracted increasing attention in recent years. Interestingly, it was found that the research direction on the degradation and removal of antibiotics has the highest number of articles compared to other two research directions, and has been increased rapidly since 2016. The environmental occurrence and the potential threats of antibiotics have contributed to the urgent need for antibiotic treatment technologies [23,24]. Meanwhile, it was also visualized with a figure for the keywords with a frequency of more than 300 times in the titles and abstracts of retrieved 49,216 papers by VOSviewer software. Plainly, all these frequently used keywords are interrelated and compatible (Figure 1b), indicating that the environmental occurrence, toxicity, degradation, and removal of antibiotics are tight connection. It can also be seen that antibiotics are emerging environmental pollutants; the toxicity and effect of antibiotics toward the environment, as well as the degradation of antibiotics has recently become a research hotspot.

Hitherto, most reported data have presented the occurrence of antibiotics in a certain environmental compartment, such as wastewater [12], surface water [25], groundwater [6], and soils [26], while information on an overview of the occurrence of antibiotics in different environmental compartments has been rarely reported. Additionally, numerous studies have investigated the toxic effects of antibiotic contaminants on aquatic organism [2,27], whereas an integral study which focusing on the non-therapeutic effects of antibiotics on the aquatic and terrestrial organisms, as well as human beings is still rare. Furthermore, most of the previous studies have investigated the distribution, effects and degradation of certain classes of antibiotics, such as TCs [8,13], FQs [14,26], and SAs [28]. However, available reviews regarding the current knowledge on a full-scale scenario of various categories antibiotics are still limited. To fully understand the influence of antibiotics on ecological environment and maintain the sustainable development of ecology, in the following we provide a comprehensive overview on the occurrence and toxicity of antibiotics in the environment, as well as treatment technologies for controlling antibiotic pollution. This study mainly focuses on six different antibiotic classes, including β-lactams, MAs, FQs, TCs, SAs and diaminopyrimidines. They were selected due to their wide application in human or veterinary medicine, frequent detection in different environmental matrixes, and potential ecotoxicity. Data on the basic physico-chemical properties of major antibiotics described in this paper is shown in Table S1. Section 2 describes the occurrence and distribution of antibiotics in different environmental compartments. Section 3 outlines available information about the toxicity effects of antibiotics on various non-target organisms and humans. Section 4 summarizes the removal and degradation methods of antibiotics, as well as their transformation products (TPs). Finally, Section 5 presents our conclusions and future research needs. The abbreviations used in this study is shown in supplemental material.

## 2. Antibiotics in the environment

**Figure 2.**
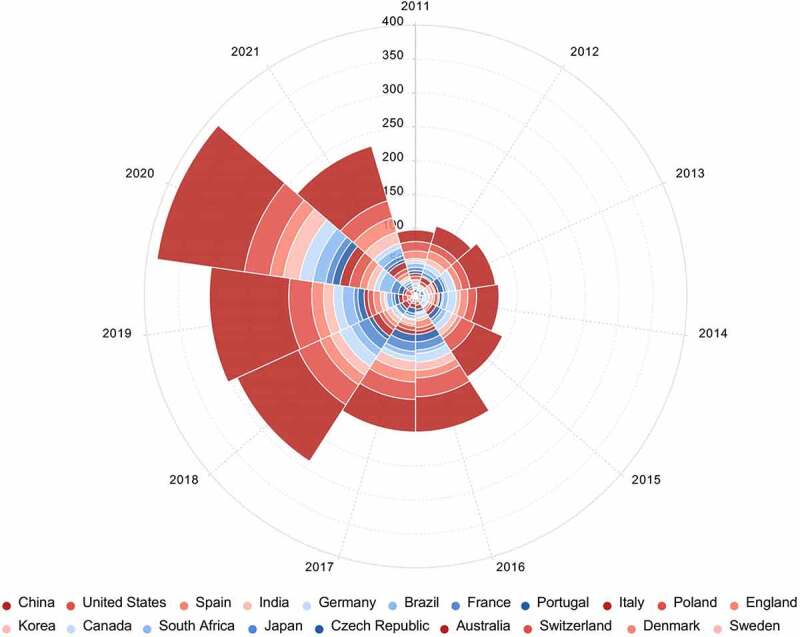
Top twenty active countries that conducted researches on the occurrence of antibiotics in the environment, and corresponding annual number of publications from 2011 to 2021

Antibiotics, as a group of pharmaceuticals are widely used in human, veterinary, aquaculture, and agriculture [2]. After use, the majority of the antibiotics eventually find their way into the environment. In recent years, many countries included Asian countries (e.g., China, India and Japan) [24,25,27], Africa countries (e.g., South Africa and Kenya) [15,29], European countries (e.g., Germany, France, and Portugal) [17,19,30], and American countries (e.g., the United States, Brazil, and Canada) [26,31] have witnessed the occurrence of antibiotics in the environment. Top 20 active countries that conducted researches on the occurrence of antibiotics in the environment, and the corresponding annual number of publications from 2011 to 2021 are shown in Figure 2. The top 20 active countries cover four countries in Asia, two countries in North America, eleven countries in Europe, one country in South America, one country in Africa, and one country in Oceania; the number of articles related to the environmental occurrence of antibiotics gradually increased during the period of 2011–2020. These results indicated that the occurrence of antibiotics in the environment has become a global issue. Additionally, the number of publications on the environmental occurrence of antibiotics in China is the highest compared to that in other countries, which may be due to that China is the largest producer, exporter and user of antibiotics in the world. Recent data (2011–2021) on concentrations of major antibiotic residues in different environmental compartments worldwide is provided in Table 1.

**Table 1.** Concentration data of frequently detected antibiotics in different environmental compartments during 2011–2021

Antibiotics	Aquatic environment (ng/L)					Solid matrices (ng/g dry matter)		Location	Refer-ence
	Influent of WWTPs	Effluent of WWTPs	Surface water (river/estuarine)	Groundwater	Seawater (harbor)	Sediment	Sewage sludge		
***β-lactams***									
Amoxicillin	2,935–6,516 (3,746)	ND-1,129						Singapore	[11]
		ND-190 (20)	5–21 (17)					Charmoise river (France)	[19]
	200–4,600	900–1,600	ND-300			ND-43.8		Kenyan	[15]
***Macrolides***									
Erythromycin			0.4–6.9 (3.9)			1.5–24.6 (10.2)		Huangpu river (China)	[21]
	610	160	ND-240			ND	ND	Msunduzi River (South Africa)	[29]
	111.4–403.3 (272.5)	89.8–112 (98.2)						Singapore	[11]
		ND-1,492 (395)	1–913 (293)					Charmoise river (France)	[19]
	3–470 (118)	ND-350 (129)					ND-100 (56)	Sweden	[55]
			32.3–2,910 (973)	ND-28.9 (5.63)				Jianghan Plain (Central China)	[48]
			2.50–8.16	ND-17.66		1.16–6.88		North Carolina (United States)	[49]
Roxithromycin			ND-227 (37.9)		ND-1.5 (0.38)			Bohai Sea (North China)	[44]
	25.2–65.2	7.82–58.0					ND-80	Guangdong (South China)	[16]
			0.2–2.2 (0.9)			0.3–4.1 (1.9)		Huangpu river (China)	[21]
			ND-173 (38.37)	ND-3.18 (0.088)				Jianghan Plain (Central China)	[48]
			ND-1.75 (0.44)			ND		Yangtze River (China)	[54]
Azithromycin		ND-88.0 (22.3)			ND-1.2 (0.14)			Bohai Sea (North China)	[44]
	1,537–2,951 (1,949)	367.3–980 (469.5)						Singapore	[11]
			221–2,819			ND-43.2		Leça River (Portugal)	[17]
Clarithromycin			ND-32.9 (5.0)		ND-0.82 (0.19)			Bohai Sea (North China)	[44]
	30.8–126	5.16–86					ND-69.5	Guangdong (South China)	[16]
	1,201–1,854 (1,497)	387.3–637.1 (531.7)						Singapore	[11]
			ND-32.1 (8.34)	ND-1.11 (0.02)				Jianghan Plain (Central China)	[48]
			ND-2.19 (0.26)			ND		Yangtze River (China)	[54]
			76.6–269			ND		Leça River (Portugal)	[17]
***Fluoroquinolones***									
Ciprofloxacin			ND-346 (101)		ND-66 (31)			Bohai Sea (North China)	[44]
	15.6–90.3	5.64–9.06					296–714	Guangdong (South China)	[16]
	2,241–6,453 (3,496)	321.3–524.1 (495.5)						Singapore	[11]
		89–3,403 (817)	5–1,523 (288)			569 ± 418		Charmoise river (France)	[19]
	83–1,406 (391)	ND-62 (38)					1,600–11,000 (4,625)	Sweden	[55]
			4.46–96.0 (25.14)	0.476–26.3 (6.23)				Jianghan Plain (central China)	[48]
			ND-0.94 (0.34)			0.28–8.43 (1.24)		Yangtze River (China)	[54]
	ND-3,000	300–2,600	ND-1,300			ND-47.4		Kenyan	[15]
			ND-339			ND		Leça River (Portugal)	[17]
Ofloxacin			ND-45.4 (9.9)		ND-6.5 (0.24)			Bohai Sea (North China)	[44]
	30.3–1,010	47.4–179					1,500–5,800	Guangdong (South China)	[16]
			ND-28.5 (6.5)			ND-12.4 (4.1)		Huangpu river (China)	[21]
		805–8,637 (4,312)	100–2,888 (1,209)			498 ± 261		Charmoise river (France)	[19]
			ND-45.3 (9.03)	ND-7.2 (0.47)				Jianghan Plain (Central China)	[48]
			ND-0.82 (0.32)			0.29–84.17 (44.27)		Yangtze River (China)	[54]
			ND-120			ND		Leça River (Portugal)	[17]
Norfloxacin			ND-572 (118)		7.5–103 (40)			Bohai Sea (North China)	[44]
	42.4–974	36.7–61					1,820–5,610	Guangdong (South China)	[16]
		97–9,347 (1,542)	14–1,261 (289)			225 ± 70		Charmoise river (France)	[19]
			9.57–277 (65.87)	1.19–142 (23.75)				Jianghan Plain (Central China)	[48]
			ND-0.83 (0.32)			0.15–0.83 (0.4)		Yangtze River (China)	[54]
	900–2,800	500–2,900	ND-2,200			ND-26.6		Kenyan	[15]
Enrofloxacin			ND-24.6 (10.6)		ND-7.6 (1.8)			Bohai Sea (North China)	[44]
			ND-14.6 (2.8)			ND-8.9 (3.2)		Huangpu river (China)	[21]
		ND-636 (57)	ND			11 ± 4.1		Charmoise river (France)	[19]
			ND-136 (35.56)	ND-30.3 (7.20)				Jianghan Plain (Central China)	[48]
			ND-0.89 (0.4)			0.31–0.95 (0.58)		Yangtze River (China)	[54]
Enoxacin			ND-508 (116)		ND-209 (62)			Bohai Sea (North China)	[44]
		ND-1,634 (169)	ND-1,310 (134)			31 ± 18		Charmoise river (France)	[19]
Lomefloxacin	3.22–25.5	ND-8.59					25.1–440	Guangdong (South China)	[16]
		ND-16 (3)	3.6–6.7 (5.5)			5.3 ± 4.9		Charmoise river (France)	[19]
			ND-36.4 (9.98)	ND-10.7 (2.11)				Jianghan Plain (Central China)	[48]
***Tetracyclines***									
Tetracycline	40.1–316	7.73–35.1					353–1,650	Guangdong (South China)	[16]
			ND-54.3 (4.2)			ND-21.7 (3.5)		Huangpu river (China)	[21]
	1,240–12,340 (3,604)	691.2–1,536 (766.4)						Singapore	[11]
		ND-238 (47)	ND-68 (16)					Charmoise river (France)	[19]
			ND-122 (36.25)	ND-38.9 (4.78)				Jianghan Plain (Central China)	[48]
Chlortetracycline	7.46–35.7	ND					34.6–455	Guangdong (South China)	[16]
			ND-46.7 (3.6)			ND-6.3 (2.4)		Huangpu river (China)	[21]
	2,333–15,911 (6,434)	1,472–1,986 (1,757)						Singapore	[11]
			12.3–109 (33)	0.71–59.6 (6.16)				Jianghan Plain, (Central China)	[48]
			ND-0.95 (0.55)			ND-0.95 (0.39)		Yangtze River (China)	[54]
Oxytetracycline	79.1–560	10.8–17.6					417–1,680	Guangdong (South China)	[16]
			ND-219.8 (78.3)			0.6–18.6 (6.9)		Huangpu river (China)	[21]
	1,629–30,049 (4,887)	839.8–2,014 (1,469)						Singapore	[11]
			ND-63.1(24.79)	ND-24.3 (3.18)				Jianghan Plain (Central China)	[48]
			ND-0.97 (0.37)			0.16–0.93 (0.41)		Yangtze River (China)	[54]
Doxycycline	ND-23.0	ND					103–232	Guangdong (South China)	[16]
			ND-112.3 (11.3)			ND-21.3 (7.0)		Huangpu river (China)	[21]
	ND-2,700	400–1,500	ND-700			ND-32.2		Kenyan	[15]
Minocycline	730.9–3,808 (1,233)	ND						Singapore	[11]
***Sulfonamides***									
Sulfamethoxazole			0.36–527 (62.8)		1.5–82 (19)			Bohai Sea (North China)	[44]
	216–239	65.2–106					ND-10.0	Guangdong (South China)	[16]
			2.2–764.9 (259.6)			0.05–0.6 (0.2)		Huangpu river (China)	[21]
	34,500	ND	ND-5,320			ND	ND	Msunduzi River (South Africa)	[29]
	893.4–1,389 (1,172)	301.5–463.4 (311.3)						Singapore	[11]
		407–12,848 (3,375)	41–3,066 (1,119)			7.2 ± 2.1		Charmoise river (France)	[19]
			1.92–30.2 (13.67)	ND-7.29 (1.01)				Jianghan Plain (Central China)	[48]
		374.3–1,309.5 (842.4)	15–218 (130)	47.8–251.4 (106)				Bolivia	[47]
			0.43–7.95 (3.84)			0.14–2.04 (1.39)		Yangtze River (China)	[54]
			5.97–14.54	1.01–54.04		2.77–6.89		North Carolina (United States)	[49]
Sulfadiazine			ND-18.7 (1.7)		ND-0.43 (0.02)			Bohai Sea (North China)	[44]
	15.2–40.1	1.14–9.67					ND	Guangdong (South China)	[16]
			4.9–112.5 (53.6)			0.07–0.71 (0.4)		Huangpu river (China)	[21]
			ND-16.7 (5.16)	ND-2.87 (0.55)				Jianghan Plain (Central China)	[48]
			ND-0.46 (0.23)			ND-0.57 (0.1)		Yangtze River (China)	[54]
Sulfamethazine	12.8–131	7.3–23.3					ND-3.83	Guangdong (South China)	[16]
			19.9–389.4 (188.9)			0.2–2.7 (1.2)		Huangpu river (China)	[21]
	ND	ND	ND-1,090			ND	ND	Msunduzi River (South Africa)	[29]
	449.9–1,814 (802.8)	73–260.8 (135.9)						Singapore	[11]
			ND-27.3 (5.36)	ND-15.9 (0.50)				Jianghan Plain (Central China)	[48]
			0.24–0.79 (0.48)			ND-0.27 (0.16)		Yangtze River (China)	[54]
Sulfapyridine	29.8–121	7.08–24.1					ND-2.31	Guangdong (South China)	[16]
			ND-103.1 (24.1)			ND-6.6 (1.7)		Huangpu river (China)	[21]
***Diaminopyrimidines***									
Trimethoprim			ND-13,600 (1,133)		1.3–330 (53)			Bohai Sea (North China)	[44]
	72.3–162	31.1–64					3.57–10.7	Guangdong (South China)	[16]
	ND	ND	ND-290			ND- 87.55	ND	Msunduzi River (South Africa)	[29]
	197.6–251.2 (235.5)	124.9–178.6 (151.6)						Singapore	[11]
		ND-5,316 (1,568)	1–1,573 (442)			52 ± 27		Charmoise river (France)	[19]
	51–340 (115)	10–130 (61)					ND-58 (42)	Sweden	[55]
		271–336.5 (304)	46–312 (159)	108–200.2 (154.2)				Bolivia	[47]
			0.24–0.96 (0.73)			ND-0.99 (0.23)		Yangtze River (China)	[54]
	100–5,600	100–500	ND-200			ND-13.3		Kenyan	[15]
			ND-110			ND		Leça River (Portugal)	[17]
***Degradation products***									
AMX penilloic acid		150		30				Israel	[133]
AMX 2ʹ,5ʹ- diketopiperazine		500						Israel	
Erythromycin -H_2_O	771–942	83.9–695					29.3–147	Guangdong (South China)	[16]
	299.3–737 (652.1)	194.5–381 (272.6)						Singapore	[11]
			ND-30 (4.67)			ND-6.78 (1.2)		Yangtze River (China)	[54]
D-ciprofloxacin		ND-127 (27)	7–71 (16)			22 ± 11		Charmoise river (France)	[19]
4-epidemeclocycline			17–1,159					Canada	[20]
Isochlortetracycline			192–3,256					Canada	
N-acetyl SMX		18–1,245 (410)	4–406 (124)					Charmoise river (France)	[19]
			ND-290.3	ND				Bolivia	[47]
SMX-N1-glucuronide			ND-233.7	ND				Bolivia	

Note: WWTPs: wastewater treatment plants; max: maximum concentration; med: median concentration; A-B (C): range (mean/median); (A ± B): (mean±SD); ND: no detected or below detection limit; AMX: amoxicillin; SMX: sulfamethoxazole; D-ciprofloxacin: desethylene ciprofloxacin

### 2.1. Occurrence of antibiotics in waste, surface, sea, ground, and drinking water

#### Wastewater

The main sources of antibiotics are wastewaters from municipalities, pharmaceutical manufacturing, and hospitals [12,24], as well as animal husbandry and aquaculture [21,27,32]. The concentrations of antibiotics in the wastewaters are provided in Table 1. Twenty-one antibiotics, including AMX, 4 MAs, 6 FQs, 5 TCs, 4 SAs, and TMP have been frequently detected in the wastewaters. Antibiotics in influents or effluents of WWTPs are at relatively high concentrations, ranging from several hundred ng/L to a few ten μg/L (Table 1). The concentrations of AMX in influents and effluents are in the range of 200–6,516 ng/L and not detected (ND) – 1,600 ng/L, respectively (Table 1), which indicates that AMX pollutant can be removed by WWTPs. The concentrations of the different antibiotic classes in influents follow the rank order SAs (ND – 34,500 ng/L) > TCs (ND – 30,049 ng/L) > FQs (ND – 6,453 ng/L) > MAs (3–2,951 ng/L), while in effluents follow the rank order SAs (ND – 12,848 ng/L) > FQs (ND – 9,347 ng/L) > TCs (ND – 2,014) > MAs (ND – 1,492 ng/L) (Table 1). Obviously, WWTPs cannot completely remove antibiotics in wastewater. Relatively high concentrations of SAs and TCs are found in WWTP influents, which may be attributed to a very high consumption of SAs and TCs in veterinary medicines and feed additives [2,5,33]. Erythromycin (ERY) is most frequently detected in effluents, ranging from ND to 1,492 ng/L; CIP (ND – 3,403 ng/L), NOR (36.7–9,347 ng/L), and ofloxacin (OFL, 47.4–8,637 ng/L) are the most frequently detected FQs in effluents; the most frequently detected TCs in effluents are TC (ND – 1,536 ng/L), chlortetracycline (CTC, ND – 1,986 ng/L), and oxytetracycline (OTC, 10.8–2,014 ng/L) (Table 1). Combination of TMP and SMX is extensively used to treat bacterial infections [34]. SMX and TMP are ubiquitously detected in the treated effluents, with the highest concentrations being 12,848 ng/L and 5,316 ng/L, respectively (Table 1). Furthermore, some degradation products are also detected in effluents, such as AMX penilloic acid, ERY-H_2_O and N-acetyl SMX, ranging from ND to 1,245 ng/L (Table 1). Overall, the most frequently detected antibiotics in effluents of WWTPs include ERY, CIP, NOR, OFL, TC, CTC, OTC, SMX, and TMP.

The occurrence of antibiotics in effluents will impact the receiving river water. Some studies have investigated the distribution of antibiotics in upstream and downstream water of WWTPs, and found that antibiotics are at higher concentrations in downstream water than that in upstream water. For example, TMP was not detected in downstream waters, but present in downstream water at high concentration (11–140 ng/L) [19,35,36]. The higher antibiotic concentrations in downstream water subjected to discharge from WWTPs than that in upstream water provide proof of the incomplete removal of antibiotics in wastewaters after treatment. Additionally, the antibiotic level in river is higher in urban than in suburban or rural areas, because of the high population density and anthropogenic activities, which result in larger inputs of wastewater into rivers [37]. Antibiotic pollution is also correlated with national income. Environmental antibiotic concentrations are prominently higher in low-income countries, where wastewater treatment operations are limited, than in high-income countries [38].

#### Surface water

Given their presence in the wastewater, antibiotics have been widely detected in the surface water. The concentrations of antibiotics in the surface water are provided in Table 1. The classes of antibiotics present in the surface water are consistent with their classes detected in the wastewater. Antibiotics in the surface water are at relatively low concentrations, ranging from ND to several thousand ng/L (Table 1). The concentrations of the different antibiotic classes in the surface water follow the rank order SAs (ND – 5,320 ng/L) > MAs (ND – 2,910 ng/L) ≈ FQs (ND – 2,888 ng/L) > TCs (ND – 700 ng/L) (Table 1). In general, the concentrations of antibiotics in the surface waters are lower than that in the wastewaters, which may be due to the effects of dilution by river water and adsorption by suspended solids and sediment [5]. 3 MAs [ERY, roxithromycin (RTM), and clarithromycin (CTM)], 3 FQs (CIP, OFL, and NOR), 3 TCs (TC, CTC, and OTC), 3 SAs [SMX, sulfadiazine (SDZ), and sulfamethazine (SMZ)], and TMP have been frequently detected in the surface waters (Table 1). The occurrence and distribution of antibiotics in the surface waters change seasonally [22,31,39]. For instance, in a study of the Jianghan Plain (China), SAs were more frequently detected in spring (wet season) than that in autumn (dry season), as in the former the seasonal rainfall facilitated the entry of antibiotics deposited on the soil surface into surface water [39]. Similar results were obtained in a study of antibiotic occurrence in the Shell Creek watershed (Nebraska, USA), where the highest concentrations of lincomycin (68 ng/L) and monensin (49 ng/L) were detected during the summer months [31]. Low flow and cold weather may also enhance the persistence of antibiotics in water. It was found that detection frequencies and concentrations of antibiotics in the surface water of the Yangtze Estuary (China) were relatively high in January [40].

#### Seawater

Marine environment is a major receptacle of terrestrial antibiotic residues deriving from wastewater effluent and river discharge [41–43]. The antibiotic concentrations in the seawaters are relatively low compared to that in the wastewaters and the river surface waters, ranging from ND to 1349.2 ng/L; ERY-H_2_O, SMX, and TMP are the most frequently detected antibiotics in the seawaters [41,42,44–46]. The lower concentrations of antibiotics in the seawaters are largely ascribed to impacts of dilution, deposition, and degradation in riverine transportation [25]. The water-exchange between the coastal waters and the open sea also impact the occurrence and distribution of antibiotics. It was found that the total concentrations of antibiotics in open bay was significantly lower than that in more enclosed bays [41,42,44]. Additionally, both antibiotic and its metabolite were detected in the seawater. In the marine samples (Liaodong Bay, China) tested by Jia et al. (2011) [43], SMX (25.2 ng/L) and its metabolite (N-acetyl-SMX, 28.6 ng/L) were the main compounds detected, suggesting that the contribution of antibiotic metabolites to environmental contamination is comparable to that of the corresponding parent compounds. This reminds us to pay more attention to the occurrence of antibiotic metabolites.

#### Groundwater and drinking water

MAs, quinolones (including FQs), TCs, and SAs were widely detected in the groundwaters, ranging from ND to a few thousand ng/L [6]. TMP residue was also prevalent in the groundwaters, with concentration ranging from 0.2 to 200.2 ng/L [30,39,47]. The distribution and concentrations of antibiotics in the groundwater are associated with seasonal variability. A previous study showed that the concentrations of FQs and TCs in the groundwater samples (Jianghan Plain, central China) were in the range of 20.2–212 ng/L and 4.99–58.9 ng/L in spring, respectively; 2.82–26.6 ng/L and 1.41–55.5 ng/L in summer, respectively; and 6.15–68.3 ng/L and 0.710–123 ng/L in winter, respectively [48]. The higher concentrations of antibiotics in spring may be attributed to the contribution of increased rainfall in spring, which could potentially lead to the recharge of groundwater by surface water [39,48]. In contrast, another study showed that the total concentrations of four antibiotics (danofloxacin, ERY, sulfamerazine, and SMX) in the groundwaters of drinking wells in the spring, autumn, and winter were in the range of ND – 5.42 ng/L, 3.57–308.04 ng/L, and 17.66–273.80 ng/L, respectively [49]. The lower concentrations of antibiotics may be ascribed to a dilution effect and greater antibiotic export, which were induced by the increased rainfall in spring [49]. Information about the occurrence of antibiotics in the drinking waters is still limited, which may be due to that detection frequencies and concentrations of antibiotics in the drinking waters were generally low. Although drinking water may contain levels of antibiotics too low to pose a direct risk to human health, indirect effects may occur due to disturbances of the microbial communities that lead to the spread of ARGs. Therefore, more investigations should be performed on the occurrence of antibiotics in the drinking waters.

In general, the concentrations of antibiotics detected in different aquatic environments range from low ng/L to a few μg/L. Different categories of antibiotics have been detected, mainly including β-lactams, MAs, FQs, TCs, SAs, diaminopyrimidines, and some metabolites (Table 1). Although the consumption of β-lactams (penicillins and cephalosporins) is high [2,10,50], in most cases, they are rarely detected in aqueous environments; which may be attributed to their easy hydrolysis and photolysis, as well as effective removals in WWTPs [15,51–53]. According to the surveys [2,5,27], MAs, FQs, TCs, and SAs were the most frequently detected classes of antibiotics in the aquatic environments, which is consistent with the most widely used categories of antibiotics [2,10,50]. Among all antibiotics tested thus far, ERY, ERY-H_2_O, CIP, NOR, TC, SMX, and TMP are the most frequently detected antibiotics in waters, with a detection typically exceeding 50%, and in some cases reaching 100% [2,11,19,21,54]. The persistence of these antibiotics in the aquatic environment could be ascribed to their good hydrophilicity, low volatility, and stability.

### 2.2. Occurrence of antibiotics in sediment, sewage sludge, biosolid, manure and soil

#### Sediment

Generally, antibiotics are detected in both waters and sediments of rivers and lakes [21,29,53,54], implying that the occurrence of antibiotics in sediments might correlate with the degree of water pollution. Concentrations of antibiotics in the sediment range from ND to a few hundred ng/g (Table 1). FQs, TCs, and SAs are the most frequently detected antibiotic families in the sediments. The total concentrations of FQs, TCs, and SAs in sediment samples range from ND to 569 ng/g, ND to 32.2 ng/g, and ND to 7.2 ng/g, respectively (Table 1). The higher concentrations of FQs and TCs may be due to that FQs and TCs show high sorption onto sediments [53]. 3 FQs (CIP, OFL, and NOR), 3 TCs (TC, CTC, and OTC), 3 SAs [SMX, SDZ, and sulfamethazine (SMZ)], and TMP have been frequently detected in the sediments (Table 1), in correspondence with their distribution in surface waters. Additionally, to the best of our knowledge, current information on the fate and transport of antibiotic residues is still limited; sediment sorption might be one of the most important mechanisms leading to persistence of antibiotic residues in the aquatic environment.

#### Sewage sludge and biosolid

In most cases, the concentrations of antibiotics in sewage sludge are higher than that in sediment; the former is in the μg/g range [16,55]. It most likely due to strong adsorption ability of sewage sludge to antibiotics. It was found that TCs and FQs were the predominant antibiotics in sewage sludge; TC and OFL had been detected in amounts up to 1,650 and 5,800 ng/g, respectively [16], indicating that the removal of TCs and FQs from sewage mainly depends on their adsorption onto sludge. Similarly, it was observed that the concentration of another FQs (i.e., CIP) adsorbed to sludge reached up to 4,625 ng/g [55]. Biosolids usually derive from treated sewage sludge and have been applied in agriculture as soil conditioners. CIP, OFL and NOR had been detected in biosolids in concentrations >1,000 ng/g [22]. Because antibiotics in sewage sludge and biosolid will be introduced into the environment if they are not further removed by other treatment process, their levels in WWTPs need to be monitored.

#### Manure and soil

Antibiotic concentrations in manures have been measured to range from trace levels to hundreds of μg/g [20,56–58]. TCs are commonly used in veterinary medicines and feed additives [23,33]. Due to the high consumption, TCs is most frequently detected in the manures. In a previous study [59], 17 antibiotics were analyzed in different animal manures (i.e., chicken, duck, pig, and cattle manure); the maximum concentration was that of OTC (416.8 μg/g) in chicken manure. Similarly, OTC, CTC and doxycycline were the frequently detected TCs antibiotics in the pig manure, with the highest concentrations being 541.020 μg/g, 392.150 μg/g, and 56.260 μg/g, respectively [33]. Additionally, concentrations of parent TCs in swine manure slurry ranged from 53 to 137 μg/L, and those of their degradation products [4-epitetracycline (4-ETC), 4-epianhydrotetracycline (4-EATC), and anhydrotetracycline] were in the range of 118–663 μg/L [20].

In most cases, antibiotics enter the soil environment following the application of antibiotic-contaminated manures. A maximum concentration of OTC of 8,400 ng/g was determined in manure-treated soils [60]. Degradation products of TCs have also been detected in manure-amended soils, with concentrations ranging from 3.4 ng/g (4-ETC) to 1,020 ng/g (4-EATC) [20]. The concentrations of antibiotic residues in soils are affected by manure source [20,58,60]. The detection frequency of antibiotic residues in different soils followed the order: poultry-manured soil > swine-manured soil > cow-manured soil [61]. Wastewater irrigation is another pathway by which antibiotics enter agriculture soils [62]. It was discovered that the levels of antibiotics and ARGs were significantly higher in irrigated soils [63]. In addition, the occurrence of antibiotics is associated with different depth layers of soil. Higher antibiotic concentration was observed in the surface soil (0–10 cm) than in deeper layers (10–40 cm) owing to their sorption and migration [20,62]. However, a previous study [61] investigated the concentrations of 13 substances in soil fertilized with animal manures, and found that concentrations of antibiotics detected in soils were higher at 20–40 cm and 40–60 cm depth than at 0–20 cm depth, implying that antibiotic residues are not easily eliminated from the deeper soil. They also found that SAs, TCs, and FQs tended to persist in deeper soil, the maximum concentrations were 1,784, 86,567, 7,220 ng/g for SMX, CTC, and CIP, respectively, at 20–60 cm soil levels.

In summary, antibiotics have been measured in concentrations ranging from ng/g to μg/g in sediment, sewage sludge, biosolid, manure, and soil [dry matter (DM)]. FQs, TCs, and SAs were most frequently detected in sediments. Concentrations of antibiotics in sewage sludge are higher than that in sediments. TCs and FQs were the predominant antibiotics in sewage sludge, due to their strong adsorption onto sludge. TCs, together with their metabolites, have been detected in manures. Soil contamination by antibiotics is associated with the application of antibiotic-contaminated manures and wastewater irrigation.

## 3. Toxicity and effects

### 3.1. Toxicity to aquatic organisms

Toxicity data (EC50 values) of commonly detected antibiotics for various aquatic organisms are shown in Table 2. Among the affected aquatic species identified thus far are non-target bacteria (e.g., *Anabaena* CPB4337 and *Vibrio fischeri*), algae [e.g., *Isochrysis galbana* and *Pseudokirchneriella subcapitata* (*P. subcapitata*)], crustaceans (e.g., *Daphnia magna*), fish (e.g., *Oryzias melastigma* and *Danio rerio*) [34,64–70]. As shown by the obtained EC50 values, the toxicity of antibiotics varies depending on test organisms and antibiotic types. However, low-trophic level species (e.g., cyanobacteria and algae) exhibit higher sensitivity to antibiotics than higher-trophic level organisms (e.g., crustaceans and fish) (Table 2); macrolide antibiotics seem to have higher toxic effects to cyanobacteria and algae compared to other antibiotic groups, with EC_50_ value < 1 mg/L [64,71]. Although the concentrations of antibiotic residues in aquatic environments range from low ng/L to μg/L, the continuous discharge and persistence of these contaminants may produce unintended effects on non-target aquatic organisms. The present review focuses on three representative taxa, including cyanobacteria, algae, and fish. These are the key taxa of great concern; due to that they occupy important trophic levels in the food chain.

**Table 2.** Toxicity data of antibiotics to non-target aquatic organisms

Antibiotics	Taxonomic group	Species	Endpoint	Value (mg/L)	Reference
***β-lactams***					
Amoxicillin	Cyanobacteria	*Anabaena* CPB4337	72 h-EC50 (bioluminescence)	56.3	[64]
	Algae	*P. subcapitata*	72 h-EC50 (growth)	>1500	
			72 h-EC50 (growth)	>2000	[86]
Ampicillin	Algae	*P. subcapitata*	72 h-EC50 (growth)	>2000	
Cephalothin	Algae	*P. subcapitata*	72 h-EC50 (growth)	>600	
***Macrolides***					
Erythromycin	Cyanobacteria	*Anabaena* CPB4337	72 h-EC50 (bioluminescence)	0.022	[64]
	Algae	*P. subcapitata*	72 h-EC50 (growth)	0.35	
Tylosin	Cyanobacteria	*A. flos-aquae*	4d-EC50 (growth)	0.098	[83]
		*S. leopoliensis*	4d-EC50 (growth)	0.096	
	Algae	*P. subcapitata*	4d-EC50 (growth)	4.41	
		*D. subspicatus*	4d-EC50 (growth)	13	
		*Chlorella vulgaris*	4d-EC50 (growth)	>86.57	
		*Navicula pelliculosa*	4d-EC50 (growth)	1.41	
		*P. tricornutum*	4d-EC50 (growth)	6.08	
		*C. closterium*	(4–5d) IC50 (growth)	0.27	[85]
		*N. ramosissima*	(4–5d) IC50 (growth)	0.99	
Clarithromycin	Cyanobacteria	*A. flos-aquae*	72 h-EC50 (growth)	0.0121	[71]
	Algae	*D. subspicatus*	72 h-EC50 (growth)	0.0371	
			72 h-NOEC	0.025	
	Duckweed	*lemna minor*	7d-EC50 (growth)	>1.9	
			7d-EC50 (growth)	0.8	
	Crustaceans	*Daphnia magna*	48 h-EC50 (immobilization)	>2	
			21d-NOEC (reproduction)	>2.1	
	Fish	*Danio rerio* (embryo)	48 h-EC50 (lethality)	>2	
***Fluoroquinolones***					
Ciprofloxacin	Marine bacteria	periphytic bacteria	72 h-EC50 (growth)	0.16	[182]
			72 h-NOEC (growth)	0.0086	
			72 h-EC50 (growth)	11.3	[86]
	Algae	*C. closterium*	(4–5d) IC50 (growth)	55.43	[85]
		*N. ramosissima*	(4–5d) IC50 (growth)	72.12	
		*Chlorella vulgaris*	72 h-IC50 (growth)	9.23	[87]
			96 h-IC50 (growth)	29.09	
			72 h-NOEC (growth)	<20	
			72-LOEC (growth)	20	
		*C. mexicana*	96 h-EC50 (growth)	65	[74]
Levofloxacin	Cyanobacteria	*Anabaena* CPB4337	72 h-EC50 (bioluminescence)	4.8	[64]
	Algae	*P. subcapitata*	72 h-EC50 (growth)	>120	
Norfloxacin	Cyanobacteria	*Anabaena* CPB4337	72 h-EC50 (bioluminescence)	5.6	
	Algae	*P. subcapitata*	72 h-EC50 (growth)	>80	
***Tetracyclines***					
Tetracycline	Cyanobacteria	*Anabaena* CPB4337	72 h-EC50 (bioluminescence)	6.2	[64]
	Algae	*P. subcapitata*	72 h-EC50 (growth)	3.31	
	Ciliates	*Stentor coeruleus*	24 h-EC50 (growth)	94.4	[69]
		*Stylonychia lemnae*	24 h-EC50 (growth)	40.1	
Oxytetracycline	Algae	*Tetraselmis suecica*	96 h-IC50 (growth)	17.25	[32]
***Sulfonamides***					
Sulfamonomethoxine	Algae	*Chlorella vulgaris*	72 h-EC50 (growth)	5.9	[65]
		*Isochrysis galbana*	72 h-EC50 (growth)	9.7	[65]
	Crustaceans	*Daphnia magna*	48 h-LC50 (survival)	48	
			21d-EC50 (reproduction)	14.9	
		*Daphnia similis*	48 h-LC50 (survival)	283	
			21d-EC50 (reproduction)	41.9	
	Fish	*Oryzias latipes*	96 h-LC50 (survival)	>1000	
Sulfamethazine	Marine bacteria	*Vibrio fischeri*	30 min-EC50	>100	[67]
		*A. globiformis*	4h-EC50	>139	
	Algae	*S. vacuolatus*	24 h-EC50 (growth)	19.52	
	Duckweed	*Lemna minor*	7d-EC50 (growth)	1.74	
Sulfadiazine	Marine bacteria	*Vibrio fischeri*	30 min-EC50	>25	
		*A. globiformis*	4h-EC50	>125	
	Algae	*S. vacuolatus*	24 h-EC50 (growth)	2.22	
	Duckweed	*Lemna minor*	7d-EC50 (growth)	0.07	
Sulfamerazine	Marine bacteria	*Vibrio fischeri*	30 min-EC50	>50	
		*A. globiformis*	4 h-EC50	>132	
	Algae	*S. vacuolatus*	24 h-EC50 (growth)	11.90	
	Duckweed	*Lemna minor*	7d-EC50 (growth)	0.68	
Sulfamethoxazole	Marine bacteria	*Vibrio fischeri*	30 min-EC50	>100	
		periphytic bacteria	72 h-EC50 (growth)	0.27	[182]
	Algae	*S. vacuolatus*	24 h-EC50 (growth)	1.54	[67]
	Duckweed	*Lemna minor*	7d-EC50 (growth)	0.21	
Sulfathiazole	Marine bacteria	*Vibrio fischeri*	30 min-EC50	>50	
	Algae	*S. vacuolatus*	24 h-EC50 (growth)	13.10	
	Duckweed	*Lemna minor*	7d-EC50 (growth)	4.89	
Sulfapyridine	Marine bacteria	*Vibrio fischeri*	30 min-EC50	>50	
	Algae	*S. vacuolatus*	24 h-EC50 (growth)	5.28	
	Duckweed	*Lemna minor*	7d-EC50 (growth)	0.46	
Sulfamethoxypyridazine	Marine bacteria	*Vibrio fischeri*	30 min-EC50	>100	
	Algae	*S. vacuolatus*	24 h-EC50 (growth)	3.82	
	Duckweed	*Lemna minor*	7d-EC50 (growth)	1.51	
***Diaminopyrimidines***					
Trimethoprim	Cyanobacteria	*A. flos-aquae*	4d-EC50 (growth)	91.68	[83]
		*S. leopoliensis*	4d-EC50 (growth)	>100	
	Algae	*P. subcapitata*	4d-EC50 (growth)	>63.37	
		*D. subspicatus*	4d-EC50 (growth)	>100	
		*Chlorella vulgaris*	4d-EC50 (growth)	>100	
		*Navicula pelliculosa*	4d-EC50 (growth)	2.14	
		*P. tricornutum*	4d-EC50 (growth)	21.66	
***Others***					
Lincomycin	Cyanobacteria	*A. flos-aquae*	4d-EC50 (growth)	0.058	[83]
		*S. leopoliensis*	4d-EC50 (growth)	0.042	
	Algae	*P. subcapitata*	4d-EC50 (growth)	3.26	
		*D. subspicatus*	4d-EC50 (growth)	7.12	
		*Chlorella vulgaris*	4d-EC50 (growth)	>100	
		*Navicula pelliculosa*	4d-EC50 (growth)	>100	
		*P. tricornutum*	4d-EC50 (growth)	>100	
		*C. closterium*	(4–5d) IC50 (growth)	14.16	[85]
		*N. ramosissima*	(4–5d) IC50 (growth)	11.08	
Chloramphenicol	Algae	*Tetraselmis suecica*	96 h-IC50 (growth)	11.16	[32]
Florphenicol	Algae	*Tetraselmis suecica*	96 h-IC50 (growth)	9.03	
Gentamycin	Algae	*P. subcapitata*	72 h-EC50 (growth)	19.2	[86]
Vancomycin	Algae	*P. subcapitata*	72 h-EC50 (growth)	724	
14-hydroxy (R)-clarithromycin	Cyanobacteria	*A. flos-aquae*	72 h-EC50 (growth)	0.0272	[71]
			72 h-NOEC	0.0027	
	Algae	*D. subspicatus*	72 h-EC50 (growth)	0.0463	
			72 h-NOEC	0.02	
	Crustaceans	*Daphnia magna*	48 h-EC50 (immobilization)	>2	
			21d-NOEC (reproduction)	>0.85	
	Fish	*Danio rerio (embryo)*	48 h-EC50 (lethality)	>2	
N-desmethyl- clarithromycin	Cyanobacteria	*A. flos-aquae*	72 h-EC50 (growth)	0.134	
	Algae	*D. subspicatus*	72 h-EC50 (growth)	0.575	
			72 h-NOEC	0.115	
	Crustaceans	*Daphnia magna*	48 h-EC50 (immobilization)	>0.7	
			21d-NOEC (reproduction)	0.15	
			21d-LOEC (reproduction)	0.75	
	Fish	*Danio rerio (embryo)*	48 h-EC50 (lethality)	>2	

**Note**: *Pseudokirchneriella subcapitata: P. subcapitata; Anabaena flos-aquae: A. flos-aquae; Synechococcus leopoliensis: S. leopoliensis; Cylindrotheca closterium: C. closterium; Navicula ramosissima: N. ramosissima; Desmodesmus subspicatus: D. subspicatus; Phaeodactylum tricornutum: P. tricornutum; Microcystis aeruginosa: M. aeruginosa; Chlamydomonas mexicana: C. mexicana; Arthrobacter globiformis: A. globiformis; Scenedesmus vacuolatus: S. vacuolatus*; EC50: median effective concentration; LC50: median lethal concentration; IC50: median inhibition concentration; NOEC: no-observed-effect concentration; LOEC: lowest observed effect concentration

Cyanobacteria and algae, as principal primary producers, are of fundamental importance in aquatic ecosystems. Therefore, toxicity effects of antibiotics on these taxa should be assessed. Generally, the toxicity studies of antibiotics in these taxa include assessments of their growth, photosynthetic capability, and antioxidant systems. Corresponding parameters involve algal cell density, algal cell size, algal biomass, exopolysaccharides content, photosynthetic pigment content (e.g., chlorophyll a, chlorophyll b, and carotenoids), and biomarkers of the antioxidant system [e.g., superoxide dismutase (SOD), malondialdehyde (MDA), catalase (CAT), glutathione reductase (GR), glutathione (GSH), and reactive oxygen species (ROS)] [34,72–75]. In most cases, adverse effects of antibiotics on physiology of cyanobacteria and algae include the inhibition of growth and the change of biomarkers activities [72–75]. For instance, it was observed that the growth and photosynthetic activity of the cyanobacterium (*Microcystis flos-aquae*) were promoted by low concentrations of ERY (0.001–0.1 μg/L), but inhibited at high concentrations (≥0.1 μg/L), possibly owing to hormesis (described by low-dose stimulation and high-dose inhibition). This effect was enhanced with exposure time increasing. Meanwhile, increasing levels of ERY induced high levels of MDA and intracellular ROS, as well as enhanced the activities of SOD and CAT [75]. Similarly, another study showed that the growth of freshwater microalgae (*Chlamydomonas mexicana*) was significantly inhibited at increased concentrations of CIP (40–100 mg/L), while MDA content and SOD activity were significantly increased [74]. Additionally, sensitivity of different microalgal species toward antibiotics varies. It was found that the growth of cyanobacterium (*Chrysosporum ovalisporum*) was significantly inhibited by FQs (ENR and NOR) (1–50 mg/L), while the growth of green algae (*Chlorella vulgaris*) was less affected by FQs. Activities of SOD, CAT, and GR in the *Chlorella vulgaris* significantly increased [73]. Microalgal species showed differential sensitivity to FQs, which may be due to their morphology, cytology, physiology, and phylogenetics [74]. Furthermore, the toxicity of a given antibiotic correlates to its property. A previous study showed that the content of GSH significantly increased in cyanobacteria (*Microcystis aeruginosa*) under 10–20 mg/L of cefradine, but decreased under exposure to NOR and AMX [72]. Previous results showed that bacterial protein synthesis inhibitors (e.g., azithromycin, florfenicol and OTC) had higher toxicity effects to green algae (*P. subcapitata*) than cell wall synthesis inhibitors (e.g., cefotaxime and AMX), most likely due to different properties of antibiotics such as electrophilicity, stability, and hydrophobicity [76].

In addition to their places at the top of the aquatic food chain, fish are a major food and nutrient source for humans. Thus, toxic effects of antibiotics on fish should be considered. Previous study showed that behavior of fathead minnow (*Pimephales promelas*) was not affected by FQs exposure [77]; however, by microscopic observation, it was found that skeletal muscle and cardiomyocyte tissues of adult zebrafish suffered severe damage when exposed to the mixture of FQs and TCs with high concentration (several tens of mg/L), imply that histopathological change in fish tissues exposed to antibiotics can be observed by microscopy [78]. Another recent study also confirmed that exposure of carp (*Cyprinus carpio*) to one of FQs (NOR: 100 ng/L and 1 mg/L) caused significant damage to the liver cells, reflecting as the enlargement of hepatocyte and cell nucleus [70]. Other adverse effects of antibiotics on physiology of fish include the change of biomarkers activities (e.g., acetylcholinesterase, SOD, CAT, MDA, glutathione peroxidase, and glutathione S-transferase), developmental toxicity (reflecting as embryo malformations, hatchability decrease, etc.), and functional disorders of nervous system [66,68,70,79,80]. Toxicity effects of antibiotic residues on the reproduction and development of fish have attracted much attention. It was observed that a mixture of β-diketone antibiotics (DKAs) had toxicity effects on both F0-zebrafish and their F1-larvae. Ovaries and testes of F0-zebrafish suffered serious damage; both estradiol in zebrafish and testosterone in male zebrafish decreased significantly following DKAs exposure. Thus, DKAs could reduce or inhibit the reproductive ability of F0-zebrafish. Also, some gene expression (*c6ast4, igfbp1b, mrpl42, tnnc2, emc4, and ddit4* etc.) of F1-larvae significantly differed in response to increasing concentrations of DKAs (control, 6.25 and 12.5 mg/L), which may result in developmental disorders or diseases of larvae [81].

To date, most studies have examined the effects of a single antibiotic on aquatic organisms, including ERY on *Microcystis flos-aquae* [75], AMX on *Microcystis aeruginosa* [82], TC on ciliates [69], NOR on *Cyprinus carpio* [70], SMZ on *Oryzias melastigma* [66], Zebrafish [68] and others [83,84]. However, because different categories of antibiotics are released into environmental matrices, aquatic organisms are always exposed to mixtures of antibiotics. Hence, with the development of analysis techniques, research interests have expanded to include investigations of combined effects of binary and pluralistic antibiotics, as well as their modes of action. Two typical models (concentration addition and response addition), together with four joint toxic effects (independent, additive, synergistic, and antagonistic effect) are currently used to evaluate toxicological interactions of mixtures [34,64,85–88]. Furthermore, not only parent antibiotics but also their TPs have toxicity effects on non-target organisms [51,71,76], implying that the potential adverse effects of TPs should not be neglected. A previous study evaluated the toxic effects of AMX and its main degradation product amoxicilloic acid (AMA); the latter likely played a principal role in toxicity test, due to that AMX was rapidly metabolized to AMA by *Cyprinus Carpio* [89]. In acute toxicity on aquatic organisms, it was found that both CTM and its metabolite 14-hydroxy(R)-CTM exhibited dramatic toxicity to cyanobacterium (*Anabaena flos-aquae*) [71]. Mixtures of SMX and its degradation products produced via ozonation [90], photodegradation [91], and persulfate oxidation [92] all exhibit comparable or higher toxicity than SMX alone in a variety of organisms (e.g., *Vibrio fischeri, P. subcapitata*, and *Daphnia magna*).

Overall, antibiotics can impact the growth, photosynthetic capability, and antioxidant systems of cyanobacteria and algae, and their toxicity correlates to its class, exposure dose, and exposure time. Antibiotics can also induce physiological changes in fish, as well as have toxicity effects on the reproduction of fish. The combined effect of binary and pluralistic antibiotics on aquatic organisms has become a focus, due to that antibiotics are often as mixtures present in the environment. Not only parent antibiotics but also their TPs have toxicity effects on non-target aquatic organisms. Currently, it remains a great challenge to identify the TPs that retain biological activity, due to the complexity and uncertainty of TPs. In the future, toxicological information for more antibiotics and their TPs, especially at environmentally relevant concentrations, as well as for more species are required to fully estimate the risks of antibiotic contamination for protecting the aquatic organisms. Additionally, precise mechanisms underlying the toxicity to aquatic organisms of antibiotics are not well understood but are increasingly being investigated in terms of proteomic responses and gene expression [70,78,81,82,93].

### 3.2. Toxicity to terrestrial plants

Antibiotics enter terrestrial plants via manure applications or wastewater irrigation, both of which may pose a risk to plant growth. Concentrations of TC and AMX accumulated in plants ranged from 4.4 to 36.8 ng/g, 13.7 to 45.2 ng/g (fresh weight), respectively, when exposing the plants (i.e., carrot and lettuce) to different concentrations (0.1–15 mg/L) of antibiotics [94]. In addition, AMX was more easily absorbed by plant alfalfa than TC, probably due to its good solubility. A previous study measured the concentration of SMZ in different parts of alfalfa and found that it was highly variable (between 0.38 ng/g in sap and 8.58 ng/g in root). The higher concentration of SMZ in the root zone could be ascribed to the direct contact between roots and SMZ solution [95].Studies have demonstrated that antibiotics may show toxic effects on farm plants (sweet oat, rice, maize, soybean etc.), owing to their bioaccumulative potential. The harmful effects include inhibition of seed germination and root elongation, changes in MDA contents and antioxidative enzyme activities (e.g., SOD, CAT and peroxidase), an increased chromosomal aberration frequency and yield reduction [96–101]. The phytotoxicity of antibiotics varies with the type of antibiotic and with the plant species. A previous study evaluated the phytotoxic effects of five antibiotics on seed germination and root elongation of crops. Obtained EC50 values of various antibiotics ranged from 10.3 to >300 mg/L; toxicity of antibiotics follows the order: TC > NOR > ERY > SMZ > CAP. The authors also found that sensitivity of crops to TC had the following decreasing order: carrot > tomato > lettuce > cucumber [99]. Although measured concentrations of antibiotics in soil or water rarely match or exceed the EC50 values, their potential toxicity should not be ignored. On the other hand, it was observed that 0.5–10 mg/L TC stimulated wheat growth (expressed as the stimulation of seed germination, cell mitotic division and seedlings growth) and had slight effects on antioxidant enzymes activity of wheat. The beneficial effect could be attributed to antisepsis effect of TC at low concentrations and the protective effects of enzymes and other cellular constituents, although the exact mechanism is unclear [102].

In summary, antibiotics can accumulate in plants. To date, further research is required to better understand the uptake and translocation of different antibiotics in more plants. Antibiotics at low concentrations can stimulate the growth of plants, while at high concentrations have harmful effects on plants growth. The stimulation mechanisms of antibiotics at low concentrations need to be further gone into.

### 3.3. Antibiotic resistance genes

Antibiotic abuse and misuse are generally described as the major reasons for antibiotic resistance [103–105]. Due to widespread use of antibiotics, antibiotic-resistant bacteria (ARB) and ARGs are abundant in human and animal feces. Following excretion, ARB enter the environment, transferring their genes to environment-indigenous microbes [106]. Additionally, the role of antibiotic-contaminated environments has gained increasing attention for the development and dissemination of antibiotic resistance. The application of antibiotic-contaminated animal manure to farmland encourages the spread of ARGs between environmental bacteria and human pathogens [107,108]. Furthermore, antibiotic residues and some contaminants (e.g., heavy metals and pesticides) in environmental matrices can support the spread of ARGs and selection of resistant bacterial populations [56,105,106,109]. On the other hand, antibiotics present in the environment can also exert selective pressure on environment-indigenous microbes persistently, inducing the uncontrolled spread of ARGs. It was confirmed that bacterial resistance to antibiotics can be obtained by biological pathways of self-formation or external stress [105].

Recently, there has been a growing concern about the spread of ARGs in the environment. A large number of ARGs have been detected in different environment compartments, mainly including wastewater, surface water, sediment, soil, and sewage sludge [36,47,54,110–113]. Wastewater is a gathering point for antibiotics, ARGs, and bacteria, and can serve as important reservoirs and environmental suppliers for ARGs [110]. A previous study investigated the occurrence of ARGs in the wastewaters, and found that various ARGs, including *bla*_TEM_, *qnr*S, *erm*B, *sul*I, and *tet*W were detected at the highest concentrations in hospital effluents and WWTP influents. The total copy numbers of ARGs ranged from 10^6^ to 10^8^ copies/mL based on Real-time PCR (qPCR) assays. The aforementioned ARGs were still present in effluents after treatment by WWTP [36]. The occurrence of ARGs in the effluents could promote the spread of ARGs in the receiving water and soil irrigated by wastewater. A previous study found that *sul*I and *sul*II genes showed high abundance in the WWTP effluent, as well as in the surface water and sediment of receiving river. Based on qPCR assays, the copy numbers of *sul*I and *sul*II in the water and sediment ranged from 9.56 × 10^4^ to 3.30 × 10^8^ copies/mL, and 5.96 × 10^4^ to 4.98 × 10^8^ copies/g, respectively [54]. Additionally, *tet* A, *tet* C, *tet* O, *sul*I, *sul*II, and *sul*III genes had high relative abundance in the irrigated soil, in corresponding to their abundance in the irrigation wastewater [63,112]. The relative abundance of *sul*I, *sul*II, *tet*M, *tet*W, and *tet*O were high in the manure and manure-amended agricultural soil [56,107], which suggested that manure can also act as an important environment supplier for ARGs. In general, tetracycline resistance genes (*tet*A, *tet*C, *tet*O and *tet*W) and sulfonamide resistance genes (*sul*I and *sul*II) genes were the most prevailing ARGs in different environmental matrixes, owing to the general use of the corresponding antibiotics, as well as their persistence in the environment [36,47,54,63,111–113].

Drinking water is a potential route of ARGs transfer into humans. Various ARGs (e.g., *sul*I, *erm*B, *tet*M, and *tet*O) was observed in drinking water source and tap water [114]. Though the relationship between ARGs in drinking water with human health risk is not very clear, consumption of drinking water polluted with ARGs may cause the dissemination of ARGs in human microbiome [115]. In addition, various ARGs (e.g., *sul*I, *amp*C, *bla*_TEM_, and *tet*G) have been found in vegetables (lettuce, carrots, and radishes) collected from the experimental land receiving animal manure as a fertilizer [107,108]. More seriously, some studies demonstrated that antibiotic-contaminated wastewater irrigated vegetables can contribute to resistance selection in human gut microbiome, which could lead to the propagation of resistance gene and resistance bacteria [116]. These findings imply that humans may be infected with ARGs via food chain. Overall, antibiotic-contaminated environments can promote the development and dissemination of antibiotic resistance. Diverse ARGs were detected in drinking water and ready-to-eat vegetables, which provided a potential route of ARGs transfer into human via water and food chain. Appropriate measures should be taken to minimize the impact of environmental stresses and antibiotic residues to ARGs dissemination, and thus protect the public health.

## 4. Degradation and removal methods

Due to the frequent detection of antibiotics in the environment and their potential threat to the ecosystem, the present review also focuses on the degradation and removal methods of antibiotics. Table 3 summarizes previous research on antibiotics and their degradation products.

**Table 3.** Identified transformation of antibiotics

**Category**	**Parent compound**	**Degradation method**	**Intermediate/product (structural formula)**	**(m/z)/[M + H]^+^**	**Proposed structure**	**Product name**	**Reaction mechanism**	**Reference**
				Experimental				
***β-lactams***	Amoxicillin	Hydrolysis (pH 2/7/8/10)	C_15_H_22_N_3_O_4_S	340.1316	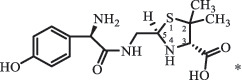 *	(5 R)-AMX penilloic acid *	β-lactam ring cleavage, decarboxylation	[132]
			C_15_H_22_N_3_O_4_S	340.1337	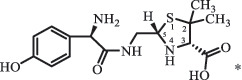 *	(5S)-AMX penilloic acid *	epimerization	
			C_16_H_20_N_3_O_5_S	366.1131	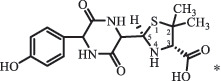 *	(5 R)-AMX 2ʹ, 5ʹ- diketopiperazine*	β-lactam ring cleavage, decarboxylation, internal rearrangement	
			C_16_H_20_N_3_O_5_S	366.1124	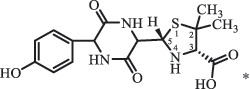 *	(5S)-AMX 2ʹ, 5ʹ- diketopiperazine*	epimerization	
			C_17_H_24_N_3_O_6_S	398.1383	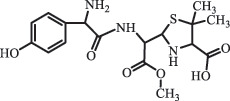	amoxicilloic acid methyl ester	β-lactam ring cleavage	
		Hydrolysis (pH 5/7/8)		189	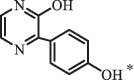 *	phenol hydroxypyrazine*	a series of AMX-degradation processes	[133]
				384	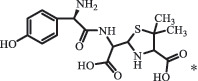 *	AMX penicilloic acid *		
	Ampicillin	Hydrolysis (pH4 & 60°C)		173.1	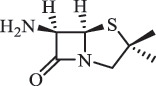		amide cleavage, ecarboxylation	[135]
				410.1	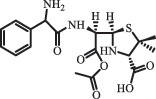		β-lactam cleavage	
		Hydrolysis (pH7/9 & 60°C)		368.1	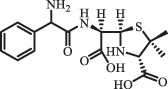		β-lactam cleavage	
				368.1	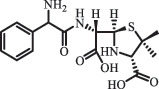		β-lactam cleavage, pimerization	
				324.1	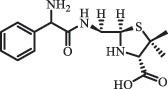		β-lactam cleavage, decarboxylation	
	Cefalotin	Hydrolysis (pH4 & 60°C)		311.1	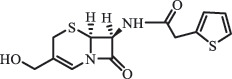		ester hydrolysis, decarboxylation	[135]
				333.1	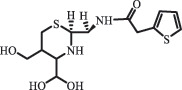		β-lactam cleavage, ester hydrolysis, decarboxylation	
		Hydrolysis (pH 7/9 &60°C)		247.0	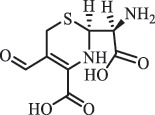		β-lactam hydration, ester and amide hydrolysis	
		Hydrolysis (pH 4/7/9 & 60°C)		377.1	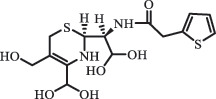		β-lactam hydration, ester hydrolysis	
				238.1	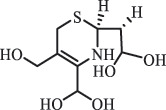		β-lactam hydration, acetate hydrolysis, C-N side chain cleavage	
				224.1	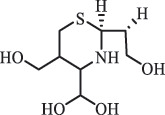		β-lactam hydration, acetate hydrolysis, C-N side chain cleavage	
	Cefoxitin	Hydrolysis (pH4/7 & 60°C)		407.0	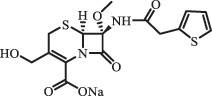		carbamate hydrolysis	[135]
				297.0	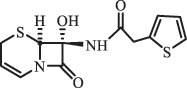		side chain cleavage, decarboxylation	
				389.0	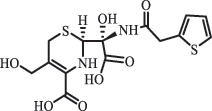		carbamate hydrolysis, β-lactam hydration	
		Hydrolysis (pH7/9 & 60°C)		221.1	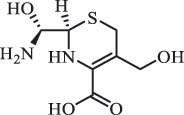		β-lactam cleavage, carbamate and amide hydrolysis	
				251.1	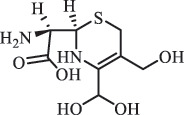		β-lactam hydration, carbamate and amide hydrolysis	
		Hydrolysis (pH4/7/9 & 60°C)		268.1	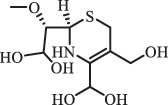		β-lactam hydration, carbamate hydrolysis, C-N side chain cleavage	
				254.1	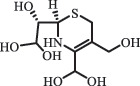		demethylation	
				236.1	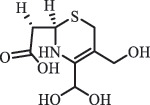		side chain cleavage	
	Cephradine	Photolysis (Simulated sunlight)		306	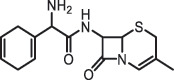		decarboxylation	[51]
				211	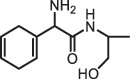		β-lactam cleavage	
	Cephalexin	Photolysis (Simulated sunlight)		304	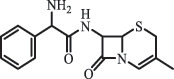		decarboxylation	[51]
				209	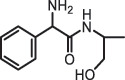		β-lactam cleavage	
	Cephapirin	Photolysis (Simulated sunlight)		126	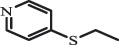			[51]
				380	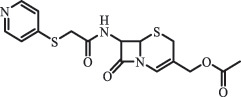		decarboxylation	
***Macrolides***	Clarithromycin	Biodegradation (*T. harzianum* and *Pleurotus ostreatus*)	C_38_H_70_NO_14_	764.48	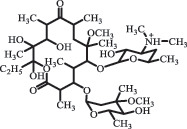	14-hydroxy-CTM	hydroxylation	[176]
			C_37_H_68_NO_13_	734.47	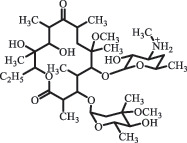	N-desmethyl-CTM	desmethyl	
			C_30_H_56_NO_11_	606.38	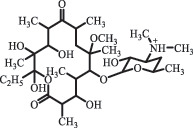	14-hydroxy-descladinosyl-CTM	cleavage of the glycosidic bond, L-cladinose moiety loss	
			C_30_H_56_NO_10_	590.39	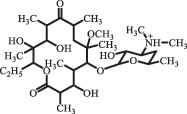	descladinosyl-CTM	cleavage of the glycosidic bond, L-cladinose moiety loss	
	Erythromycin	UV photolysis (254 nm)	C_34_H_64_NO_12_	678.4429	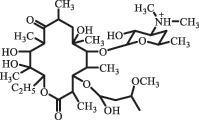		cladinose ring cleavage	[152]
			C_29_H_54_NO_12_	608.3647	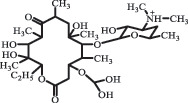		cladinose ring cleavage	
			C_29_H_52_NO_11_	590.3542	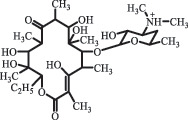		cladinose ring cleavage	
			C_23_H_41_O_7_	429.2825	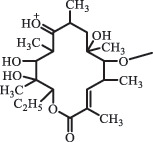		formate adduct	
		Biodegradation (*Ochrobactrum* sp. strain)		575.37	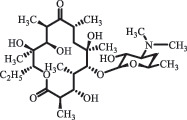	3-depyranosyloxy erythromycin	depyranosyloxy	[175]
				418.26	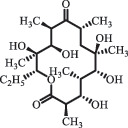	7,12-dyhydroxy-6-deoxyerythronolide		
				386.27	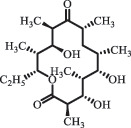	6-deoxyerythronolide		
				75.04	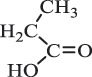		propionaldehyde	
	Roxithromycin	UV photolysis (254 nm)	C_37_H_68_NO_13_	734.4691	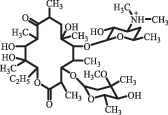	erythromycin		[152]
			C_29_H_54_NO_10_	576.3748	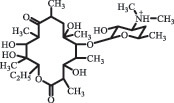		cladinose moiety loss	
	Tylosin	Hydrolysis (pH9 & 60°C)		936.6	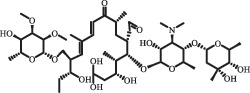		cyclic ester hydrolysis, alcohol formation	[136]
				934.5	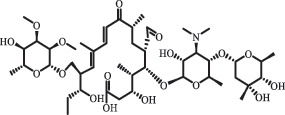		cyclic ester hydrolysis	
				916.5	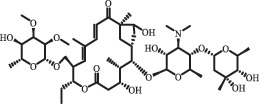		tylosin isomer	
		Hydrolysis (pH4 & 60°C)		772.4	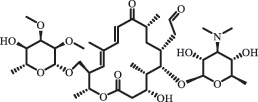		mycarose loss	
	Spiramycin	Hydrolysis (pH7/9 & 60°C)		861.5	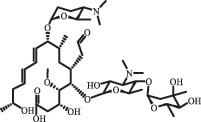		cyclic ester hydrolysis	[136]
		Hydrolysis (pH4 & 60°C)		699.4	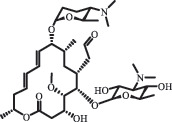		mycarose sugar loss	
				558.3	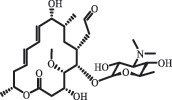		mycarose and forosamine loss	
***Fluoroquinolones***	Enrofloxacin	Photolysis (pH 7–8 & Simulated sunlight)	C_19_H_23_N_3_O_4_	358.1769	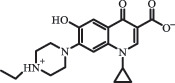		hydroxyl group substitutes F atom	[139]
			C_17_H_21_N_3_O_3_	316.1660	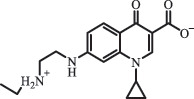		defluorination, piperazine ring cleavage	
			C_17_H_18_FN_3_O_3_	332.1415	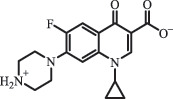	ciprofloxacin	desethyl	
			C_19_H_23_N_3_O_5_	374.1713	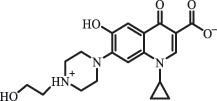		hydroxylation	
	Ciprofloxacin	Photolysis		330	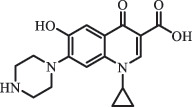	1-cyclopropyl-6-hydroxy-4-oxo-7-(piperazin-1-yl)-1,4-dihydroquinoline-3- carboxyl acid	hydroxyl group substitutes F atom	[141]
				346	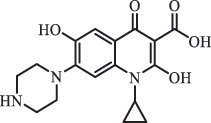	1-cyclopropyl-2,6-dihydroxy-4-oxo-7-(piperazin–yl) 1,4-dihydroquin oline-3-carboxyl acid	di-hydroxylation	
				316	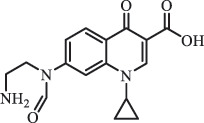	7-[(2-ethylamino)(formyl)amino]-1-cyclopropyl-4-oxo-1,4-dihydroquinoline-3- carboxyl acid	defluorination, piperazine ring cleavage	
				288	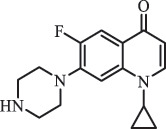	1-cyclopropyl-6-fluoro-7- (piperazin-1-yl) quinolin- 4(1H)-one	decarboxylation	
		UV photolysis (254 nm)	C_17_H_19_FN_3_O_4_	348.1354	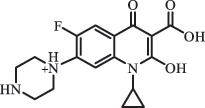		mono-hydroxylation	[152]
			C_17_H_20_FN_3_O_5_	346.1394	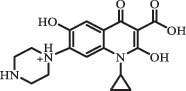		di-hydroxylation	
			C_17_H_20_FN_3_O_4_	330.1444	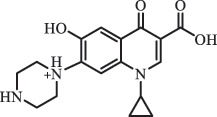		hydroxyl group substitutes F atom	
			C_16_H_18_FN_3_O_4_	316.1287	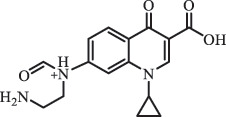		piperazinyl ring cleavage	
			C_15_H_18_FN_3_O_2_	272.1391	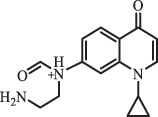		piperazinyl ring cleavage, decarboxylation	
			C_15_H_18_FN_3_O_3_	288.134	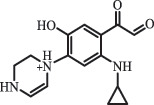			
			C_17_H_20_FN_3_O_3_	314.1499	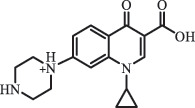		defluorination	
			C_13_H_13_FN_2_O_3_	245.0917	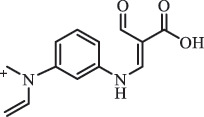		piperazinyl ring cleavage	
		Biodegradation (mixture of F11, FP1 and S2)	C_13_H_12_FN_2_O_3_	262.90	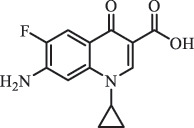		piperazine ring cleavage	[165]
			C_15_H_17_FN_3_O_3_	305.93	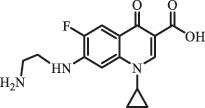		C_2_H_2_ loss	
			C_17_H_19_FN_3_O_4_	347.90	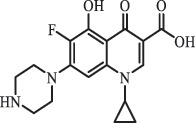		hydroxylation	
	Norfloxacin	UV Photolysis (pH 2–12)	C_16_H_12_FN_2_O_7_	363.0631	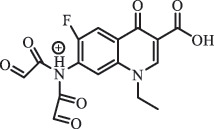		oxidation	[137]
			C_14_H_17_FN_3_O_3_	294.1237	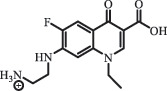		piperazine ring cleavage	
			C_14_H_16_FN_2_O_3_	279.1132	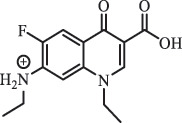			
			C_12_H_12_FN_2_O_3_	251.0827	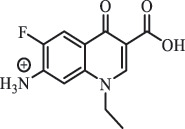			
			C_16_H_15_FN_3_O_5_	348.1013	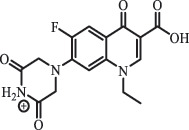		oxidation	
		Biodegradation (mixture of F11, FP1 and S2)	C_16_H_19_FN_3_O_4_	335.90	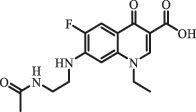		piperazinyl ring cleavage	[165]
			C_14_H_17_FN_3_O_3_	293.93	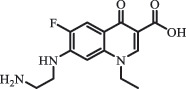		piperazinyl ring cleavage (C_2_H_2_ loss)	
			C_16_H_20_N_3_O_4_	317.93	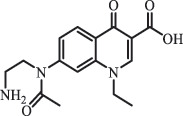		piperazine ring cleavage, amide formation, defluorination	
	Ofloxacin	Sorption on TiO_2_ surfaces		227.0	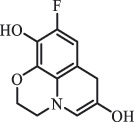		piperazinyl ring loss, demethylation	[183]
				242.9	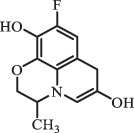		piperazinyl ring loss, decarboxylation, hydroxylation	
				259.0	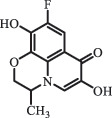		piperazinyl ring loss, decarboxylation, hydroxylation	
		Desorption from TiO_2_ surfaces		159.0	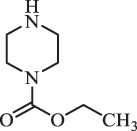			
				319.9	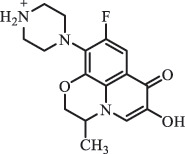		demethylation	
				348.1	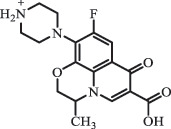		demethylation	
		Biodegradation (mixture of F11, FP1 and S2)	C_17_H_19_FN_3_O_4_	348.00	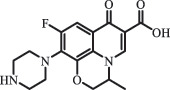		demethylation	[165]
			C_16_H_19_FN_3_O_4_	336.10	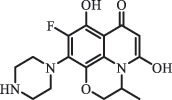		decarboxylation, hydroxylation	
	Moxifloxacin	Biodegradation (mixture of F11, FP1 and S2)	C_14_H_14_FN_2_O_4_	292.90	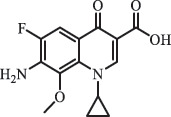		(4aS,7aS)-octahydro-6 H- pyrrolo [3,4-b] pyridine-6-yl group oxidation	[165]
			C_21_H_23_FN_3_O_5_	415.92	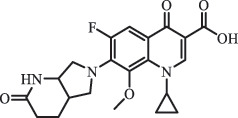			
***Tetracyclines***	Oxytetracycline	UV-visible light photolysis		416.5	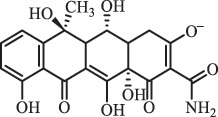	analogue of 4-dedimethylaminotetrcycline (DTC)	dedimethylamino	[138]
	Chlortetracycline	Biodegradation (*Firmicutes, Proteobacteria* and *Bacteroidetes*	C_18_H_20_O_6_	332.1	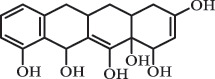		Cl atom loss, -N(CH_3_)_2_ loss	[177]
			C_22_H_23_N_2_O_8_	445.1	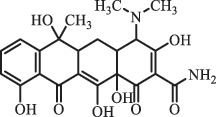	tetracycline	Cl atom loss	
			C_21_H_21_ClN_2_O_8_	465.0	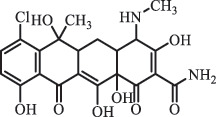	6-demethyl- chlortetracycline	methyl group loss from the -N(CH_3_)_2_	
				511	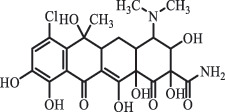		di-hydroxylation	
***Sulfonamides***	Sulfadiazine	Biodegradation (*Arthrobacter* spp)		96	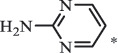 *	2-Aminopyrimidine*		[171]
				112	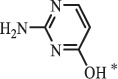 *	2-amino-4- hydroxypyrimidine *	hydroxylation	
				128	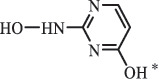 *	2-amino-4, 6-dihydroxypyrimidine*	di-hydroxylation	
				173	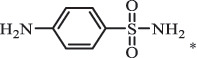 *	Sulfanilamide*		
				87	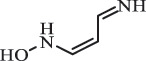			
				176	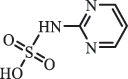		pyrimidin-2ylsulfamic acid	
					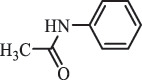		aniline acetylation	
	Sulfanilamide	Biodegradation		108.1	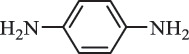		*p*-phenylenediamine	[180]
				155.8	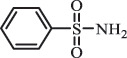		benzene sulfonamide	
				190.4	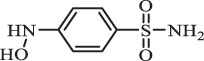		hydroxylamine benzene sulfonamide	
	Sulfamethoxazole	UV photolysis (350 nm)	C_10_H_14_N_3_O	192.1131	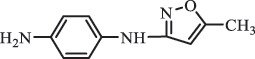		sulfur dioxide loss	[152]
		Biodegradation	C_12_H_13_N_3_O_4_S	294.0554	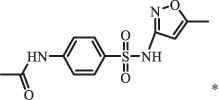 *	N4-acetyl -SMX*	acetylation	[184]
			C_12_H_13_N_3_O_4_S	296.0696	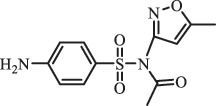	N1-acetyl – SMX	acetylation	
	Trimethoprim	Biodegradation	C_14_H_18_N_4_O_4_	307.1399	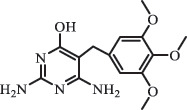		hydroxylation	[184]
	Sulfamethoxypyridazine	UV photolysis	C_11_H_12_N_4_O_4_S	297.1	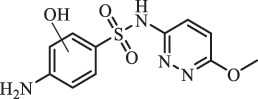		hydroxylation	[151]
			C_11_H_11_N_4_O_4_S	295.1	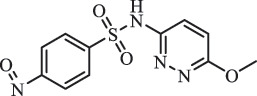		N-oxidation	
***Others***	Chloramphenicol	Hydrolysis (pH4/7/9 & 60°C)		213.1	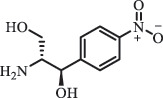		amide hydrolysis	[136]
	Florfenicol	Hydrolysis (pH4/7/9 & 60°C)		356.0	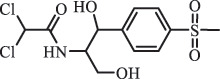		alkyl fluorine hydrolysis	[136]
		Hydrolysis (pH7 & 60°C)		288.1	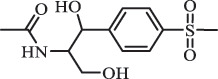		dechlorination and alkyl fluorine hydrolysis	
		Hydrolysis (pH4 & 60°C)		248.1	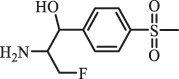		amide hydrolysis	
		Hydrolysis (pH7/9 & 60°C)		272.1	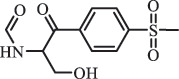		alkyl fluorine hydrolysis, dechlorination, demethylation	
				246.1	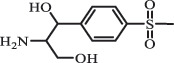		Amide and alkyl fluorine hydrolysis	
	Clindamycine	Biodegradation	C_18_H_33_ClN_2_O_6_S	441.1817	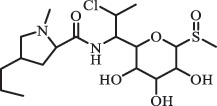		clindamycin sulfoxide	[184]
			C_18_H_33_ClN_2_O_7_S	457.1784	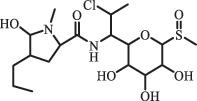		Hydroxy clindamycin sulfoxide	
			C_17_H_31_ClN_2_O_5_S	411.1713	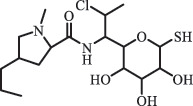		S-demethyl clindamycin	
			C_17_H_31_ClN_2_O_5_S	411.1720	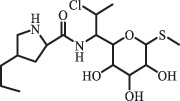		N-demethyl clindamycin	

*Indicates exact structure.

### 4.1. Adsorption

Adsorption plays a key role for the removal of antibiotics present in the natural environment. Many studies have described the adsorption of antibiotics on soil [117], sediment [118], and natural minerals (e.g., goethite and sepiolite) [119,120]. The adsorption behavior of antibiotics in soils is related to physical-chemical properties of soils (e.g., pH value, ion strength, and organic matter) and antibiotic species [117]. For example, it was found that illite-containing soils have higher sorption capacity for OTC, but loosely binds to OTC; whereas soils containing organic matter and kaolinite have lower sorption capacity, but tightly binds to OTC, implying that organic matter and clay type significantly impact OTC adsorption [121]. In general, based on the sorption coefficient values (Kd), the adsorption of antibiotics to soils follows the order: TCs > FQs > MAs > SAs, owing to the difference of their structures and functional groups [117]. It was confirmed by the fact that Kd values of representative antibiotics TC, NOR, ERY, SMZ in soils were 1093, 591, 130, 1.37 L/Kg, respectively [122].

Additionally, carbon-based adsorbent has been considered as an effective material for antibiotic wastewater treatment due to its cost-effectiveness, high efficiency and environmental friendliness [28,123–125]. It was found that ZnCl_2_ modified biochar showed efficient adsorption capacity for TC removal. The adsorption mechanism was ascribed to electrostatic interaction, pore filling, π-π conjugation and H bonding [126]. Furthermore, many carbon-based adsorbents have been used for treatment of different groups of antibiotics, such as activated carbon for TCs, quinolones and penicillins adsorption [23], reduced graphene oxides for SAs adsorption [127], multi-walled carbon nanotubes for CAPs and SAs adsorption [128], biochar for SAs adsorption [129]. The aforementioned studies suggested that interactions between carbon-based adsorbents and antibiotics showed great potential in the mitigation of antibiotic contamination.

In recent time, many studies have mainly focused on the adsorption performance, as well as the mechanisms and factors influencing adsorption of antibiotic contaminants onto the adsorbents, while few studies have dealt with issues related to the recovery and regeneration of adsorbents. In reality, these used adsorbents often contain a high level of antibiotics. For example, when the activated carbon was employed as an adsorbent, the adsorption capacities for TCs, quinolones, and penicillins were up to 1340.8, 638.6, and 570.4 mg/g, respectively [23]. The discharge of used adsorbents without adequate treatment may lead to a secondary pollution in the environment. Various techniques have been employed in the regeneration of carbon-based adsorbents, such as steam regeneration, thermal regeneration, solvent regeneration (regeneration of inorganic chemicals and organic solvent regeneration), microwave irradiation regeneration, supercritical fluid regeneration, wet oxidation regeneration, electrochemical regeneration, and bio-regeneration [130,131]. Among these processes, antibiotic contaminants in the spent adsorbents can be decomposed in the processes of thermal regeneration, microwave irradiation regeneration, wet oxidation regeneration, and electrochemical regeneration. In the bio-regeneration process, antibiotic contaminants could be degraded into small intermediates by microbes, which will eventually be converted into CO_2_ and H_2_O. To protect the environment and achieve sustainable development, further studies are still needed to investigate the adsorption performance and regeneration of adsorbents simultaneously.

### 4.2. Hydrolysis

Hydrolysis is an important degradation pathway for some organic substances, especially for amides and esters. Temperature and pH value are the main factors that contributed to the hydrolysis of antibiotics. AMX, one of β-lactam antibiotics, is unstable and easily degrades in aqueous systems due to hydrolysis in its β-lactam ring. AMX penilloic acid and AMX 2ʹ, 5ʹ- diketopiperazine are two major hydrolysis products of AMX [132,133]. It was found that AMX penilloic acid, AMX 2ʹ, 5ʹ-diketopiperazine, and AMX penicilloic acid were unstable and they could be further degraded to generate 23 TPs in solutions of different pH conditions. Some stable TPs from above three AMX hydrolysis products were supposed to be penicillamine disulfide, dehydrocarboxylated AMX penilloic acid and 2-[amino(carboxy)methyl]-5,5-dimethyl-1,3-thiazolidine-4-carboxylic acid [134]. Hydrolysis half-life is often used to describe hydrolysis rate. Hydrolysis half-lives of cefalotin, cefoxitin and ampicillin were 5.3, 9.3, and 27 d under ambient conditions (pH 7 & 25°C), respectively. When increasing temperature (pH 7 & 60°C), the half-lives for the same compounds were 0.067, 0.11, and 1.1 d, respectively. These results indicate that hydrolyzation is highly temperature-dependent. Suggested degradation products likely derive from the hydrolysis of ester, carbamate and amide moieties [135]. Further confirmation is obtained from the hydrolysis of CAP, florfenicol, spiramycin and tylosin [136].

### 4.3. Photodegradation and oxidization

Photodegradation is a universal decomposition pathway for organic pollutants, including direct photodegradation, sensitized photodegradation, and photo-oxidation process. The photolysis process of antibiotics is affected by many factors, e.g., water composition (such as inorganic compounds, kinds and content of dissolved organic matter), water property (pH and temperature), photosource, photocatalyst, structure and property of organic pollutants. For instance, some antibiotics (e.g., NOR and OTC) exist in various forms owing to protonation in aqueous solutions of different pH value, which may affect their absorbance and result in differences in photolysis kinetics [137,138].

FQs are sensitive to light. Direct photolysis rates of FQs were affected by pH due to that they can exist in various forms (cationic, zwitterionic, or anionic form) relying on solution pH [139,140]. The fastest degradation occurred at neutral or slightly alkaline condition under simulated sunlight irradiation due to the dominance of the zwitterionic FQs. Photoproducts of NOR and OFL were ineffective, however, ENR photoproducts retained significant activity by *Escherichia coli* DH5α growth inhibition assay [139]. The activity of photoproducts from ENR obtained by Ge’s study [140] is consistent with those reported by Wammer’s study [139]. They further discovered the half-lives of six FQs undergoing solar-mediated direct photodegradation ranged from 0.56 min to 28.8 min in surface waters. Photosensitizers [e.g., humic substances (HSs)] are proved to accelerate photolysis under radiation exposure. In Porras’s study [141], they found that combination of HSs with CIP can facilitate photodecomposition of CIP in neutral and alkaline solutions; in the presence of HSs, the half-life of CIP (40 min < t_1/2_ < 60 min) was significantly decreased compared to CIP alone (t _1/2_ = 2 h); however, HSs did not change the photodegradation routes. The photolysis products of CIP were generated through substitution of fluorine atom (F) by hydroxyl (-OH), di-hydroxylation, oxidation of piperizine ring subsequent to defluorination and decarboxylation deriving from parent compound. The authors also found that the antimicrobial activity of CIP, assessed by *Staphylococcus aureus* and *Escherichia coli* inhibition assay, decreased significantly after irradiation in the presence of humic acid (HA), whereas only decreased slightly by direct photolysis. FQs removal via photo-oxidation process can also be an effective option, due to their piperazine moiety susceptible to electrophilic attacks by the strong oxidizing radicals (e.g., hydroxyl radical and sulfate radical), which were produced by the photolysis of oxidants (e.g., H_2_O_2_, persulfate, and peroxymonosulfate); furthermore, the toxicity of FQs and obtained products decreased significantly [142–144]. For example, the removal efficiency of CIP was about 80% in the medium pressure UV-activated peroxymonosulfate system; hydroxyl radical and sulfate radical made great contributions to the degradation of CIP; the genotoxicity of CIP solutions decreased after treatment [144].

Photolysis is the main elimination pathway of TCs [145]. In laboratory test, Jin’s team investigated the direct photolysis of OTC under UV-visible light irradiation and oxygen-free conditions. They found that the photolysis rate of OTC decreased as the initial concentration increased; but enhanced as the temperature rose. They further discovered the photolysis behavior was pH-dependent, and the half-lives of OTC ranged from 22 to 194 min at different pH values (pH 9.2–1.4). Alkaline conditions were more conducive to OTC photolysis than acid and neutral conditions, likely related to inter-/intramolecular proton transfer in the excited states [138]. Photolytic degradation was carried out combined with HA [145]; the authors found that HA accelerated the photolysis of TCT under UVA irradiation due to its photosensitization effect, whereas it inhibited TiO_2_ photocatalytic process by TiO_2_ surface deactivation and hydroxyl radical (•OH) quenching. The photodegradation of TC followed pseudo-first-order kinetics, via photodegradation pathways that may include N-demethylation, hydroxylation and H-abstraction. TCs can also be removed by photo-oxidation process. It was found that 82% of TC was degraded in the medium pressure UV/peroxymonosulfate system; hydroxyl radical dominated the degradation of TC; the proposed transformation mechanisms included hydroxylation, demethylation, and decarbonylation of TC [146].

**Figure 3.**
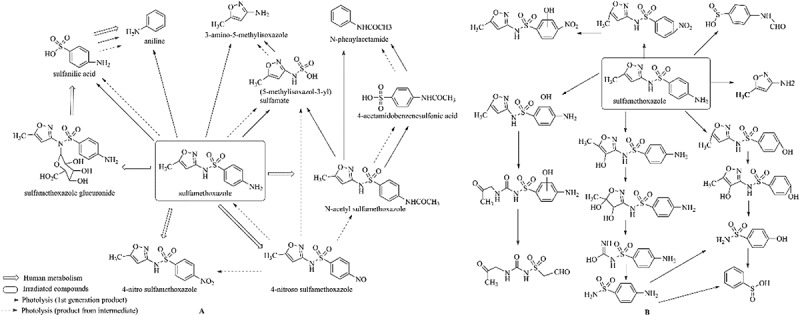
Proposed degradation pathways for SMX and its metabolites. (a) Under simulated solar (source [148]:); (b) under UV/CoFe_2_O_4_/TiO_2_ (source [150]:)

SAs are hydrolytically stable in aqueous solutions even at an elevated temperature, but sensitive to light [28,147–149]. A previous study showed that the degradation degree of SAs was 88% – 98%, except SMZ (52%) under simulated sunlight irradiation [147]. Regarding the photostability of SMX and its metabolites, both of them can undergo photolysis under simulated solar irradiation. Cleavage of the sulfonamide bond and SO_2_ extrusion were considered as major degradation pathways. Figure 3a illustrates suggested degradation pathways of SMX and its metabolites. Interestingly, the authors also found that a small amount of 4-nitroso-SMX (NO-SMX) can be transformed back to SMX [148]. The photodegradation of SMX under UV radiation with photocatalyst CoFe_2_O_4_/TiO_2_ has been described [150], the proposed pathway is shown in Figure 3b. In Figure 3, we can find that the photolysis pathway of the same antibiotic may differ in response to a different light source. Additionally, one of SAs (sulfamethoxypyridazine) was degraded completely within 128 min under UV irradiation and the hydroxylation might be the main photolysis pathway [151]. The results obtained by Lian’s team [149] showed that the degradation of SAs followed pseudo-first order reaction kinetics on UV irradiation (254 nm); corresponding kinetic parameters [i.e., molar absorption coefficients (at 254 nm), fluence-based photolysisrate constants and quantum yield] were investigated. The SAs with a penta-heterocycle (e.g., SMX and sulfathiazole) showed a higher photodegradation rate compared to those with a hexa-heterocycle (e.g., SMZ and SDZ). The photolysis of MAs has also been described. It was found that upon exposure to simulated sunlight, RTM and ERY degraded with half-life of 2.4–10 d. RTM can be changed to ERY with the illumination of UV light (254 nm); the proposed mechanism was the hydrolysis of imine group to produce carbonyl group [152].

In natural environment, sunlight plays an important role in eliminating antibiotics present in waters. Using the natural sunlight to remove antibiotic contamination is promising owing to its low cost and high efficiency. The photodegradation of antibiotics by natural or artificial sunlight has been the subject of research. For example, Sturini’ group [120,153–157] has been working on photodecomposition of FQs (e.g., CIP, ENR, and OFL) under natural/simulated sunlight. They found that solar light effectively degraded FQs, but the photodegradation products retained significant biotoxicity. The study on applying sunlight to remove OTC showed that the outdoor half-life of the antibiotic in midsummer ranged from 21 to 25 min, what’s more, OTC photodegradation can be enhanced in alkaline condition combining with sea salt [158]. Upon exposure to artificial sunlight for 48 h, SMX (dissolved in lake surface water) can generate eight photo-TPs [159]. Currently, photolysis for the removal of antibiotics is being intensively researched but in many cases the toxicity of the photoproducts remains to be determined. Oxidation is also important for the removal of antibiotic pollutants in the natural environment. A previous study showed that SA, TC, and quinolone antibiotics can be removed simultaneously via laccase-mediated oxidation combined with soil adsorption. The removal rates of each antibiotic in 15 min were > 70% and reached approximately 100% after 180 min under optimum conditions [160].

### 4.4. Biodegradation

Microbial degradation serving as one major process for the removal of antibiotics is of growing interest. The degradation process is affected by many factors, such as microbial species, anaerobic and aerobic conditions, other readily biodegradable substrates, concentration of targeted antibiotics, precipitation, and temperature.

In conventional municipal WWTPs, FQs are not easily biodegradable. It was confirmed by different treatments such as anaerobic, anoxic, and aerobic treatment. Mass change percentages of FQs were extremely low, ranging from −33% to 22%; the dewatered sludge contained 50–87% of the initial FQs [161]. However, FQs can be effectively biodegraded when treated with specific microbial community [162–166]. Through the combining use of *Labrys portucalensis* F11, *Rhodococcus* sp. FP1, and *Rhodococcus* sp. S2, biodegradation rate of OFL, NOR, CIP, and moxifloxacin (10 mg/L) at the 19th day was 98.3%, 96.1%, 94.7%, and 80.5%, respectively, indicating that microbial alliance performed great degradation ability even exposed to high level of FQs [165]. 40% – 55% of ENR (2–3 mg/L) was degraded when treated with the microbial inocula obtained from rhizosediment of plants; the main biodegradation intermediates were CIP and NOR; the relative abundance of phyla Proteobacteria (e.g., *Achromobacter* genus) and Bacteroidetes (e.g., *Dysgonomonas* and *Flavobacterium* genera) in the microbial inocula significantly increased after treated with ENR [164]. A thermophilic bacterium (*Thermus thermophilus*, designated strain C419) isolated from sludge can be used for FQs degradation. 51.45% of CIP (5 mg/L) was removed after 120 h of incubation with strain C419. Regarding other FQs, after a 72 h of incubation with strain C419, the removal efficiency of ENR, OFL, and NOR (5 mg/L) was 74%, 70%, and 63%, respectively. The antibacterial activity of FQs (CIP, ENR, OFL, and NOR) decreased after treated with the strain C419 [166]. The fate and transformation of antibiotic residues in different environmental matrixes can be explored using radioactive antibiotics. It was found that no mineralization of ^14^C-CIP in water was observed, while 0.9% mineralization was observed in soil after 93 days. The mineralization of ^14^C-CIP in sterile soil was decreased to about 0.4%, which indicated that biotic process can contribute to the degradation of CIP. Non-extractable residues of ^14^C-CIP in soil on day 0 and day 93 accounted for 57% and 88%, respectively, indicating CIP strong sorption onto soil [167]. The sorption of antibiotics in soil could enhance their persistent in the environment, which may reduce the bioavailability of antibiotics.

By contrast, some SAs are easily biodegradable [168–170]. For example, the degradation rates of SMX, sulfadimethoxine, and SMZ in sludge were 99.8%, 96.6%, and 97.8% on 82th day, respectively. The major bacterial communities were *Acinetobacter* and *Pseudomonas*, which made great contributions to the degradation of SAs in sludge [169]. Some bacterial strains isolated from activated sludge can be applied to degrade SDZ, including genus *Arthrobacter* (strain D2 and strain D4), *Paracoccus, Methylobacterium*, and *Kribbella*; the degradation rates of SDZ by microorganism were in the range of 50–99.8% depending on the microbial species; the principal intermediate products in SDZ biodegradation were 2-aminopyrimidine and 4-hydroxy-2-aminopyrimidine [171,172]. Though SAs are biodegradable, some, but not all of the TPs of SAs are biodegradable. It was found that TPs of SMX (i.e., 3-amino-5-methylisoxazole (3A5M), 4-nitro-sulfamethxoazole (NO_2_-SMX), SMX isomer and [C_10_H_13_N_3_O_4_S]) were biodegradable, whereas sulfanilic acid and 5-methylisoxazol-3-yl-sulfamate mainly exhibited abiotic attenuation [159]. Interestingly, SMX concentration increased when the irradiated solution was incubated, which is in accordance with a previous work [148]; it was likely due to back-transformation of NO_2_-SMX by microorganism. It was found that SMZ can be eliminated by plant *Tripolium pannonicum* and further under anaerobic digestion, which suggested that *Tripolium pannonicum* has a potential ability for treating wastewater [173]. Additionally, radioactive antibiotics can be used to explore the fate and transformation of antibiotics. A previous study showed that the maximum mineralization rate of ^14^C-SMX was 3%. The bioavailable SMX fraction slightly decreased from 98% to 94%, while the non-extractable fraction in the sludge slightly increased from 1% to 3.4% under aerobic heterotrophic conditions. These results indicated that the biotransformation was responsible for the removal of SMX, while mineralization and sorption were less important [174]. With the exception of FQs and SAs, other antibiotics are also biodegradable, such as ERY [175], CTM [176], and CTC [177]. The removal of ceftiofur, a representative cephalosporin, can reach 100% in inoculated culture medium and is independent of its concentration as well as the concomitant presence of ENR [164].

Compared to other abiotic removal methods, biodegradation has its advantages of cost-effectiveness and environmental friendliness in actual application. Considering the actual application situation, current focuses of research interest gradually pay attention to the identification and structural analysis of effective antibiotic-degrading strains and the biodegradation capability of selected microbial community to antibiotics [169–172,175–180]. On the other hand, the structure and diversity of microbial communities can be changed, as the resident species become acclimated to the target antibiotics [164]. As the authors shown, a predominant selection of microorganisms belonging to the phyla Proteobacteria and Bacteroidetes are associated with the biodegradation of ceftiofur and ENR. Therefore, future researches should pay more attention to the screening of highly efficient antibiotic-degrading bacterial strains from the environmental matrices (e.g., sludge, sediment, manure, and soil) containing antibiotic residues, which may stimulate the removal of antibiotics from contaminated environments. Currently, metagenomics approaches could be a powerful tool to understand the microbial community structure and functioning [93,181].

## 5. Conclusions and future perspectives

Antibiotics, as emerging contaminants, were found in various environmental compartments of different regions. The concentrations of antibiotic residues were ranging from low ng/L to a few μg/L in aquatic environments, and from ng/g to μg/g in solid matrices. Among all of antibiotic classes, MAs, FQs, TCs, and SAs were the dominant antibiotic groups in the environment. The frequently detected antibiotics were ERY, ERY-H_2_O, CIP, NOR, TC, SMX, and TMP. The factors affecting antibiotic occurrence include antibiotic consumption, national income, population density, wastewater treatment technology, and seasonal rainfall. Both antibiotics and their TPs have toxicity effects on non-target organisms in the aquatic and terrestrial ecosystems. The occurrence and spread of ARGs/ARB in the environment are of growing interest, owing to their potential risks to human health. Studies of the degradation and removal of antibiotics have shown that β-lactams can be hydrolyzed; FQs and SAs can undergo biodegradation by specific microbes; and FQs, TCs, and SAs are most effectively removed by photolysis and oxidation. With the development of LC-MS/MS methods, research interests have expanded to elucidate TPs of antibiotics, their activity, and their fates in the ecosystem.

Given the extensive use and continuous discharge of antibiotics, there is a need for more researches on the environmental occurrence, toxicity, degradation and removal of antibiotics. To achieve the sustainable development, the future research directions are suggested as follows. (1) The development of analytical methods for the detection of a greater number of antibiotics and their TPs, in order to improve risk assessments and establish quality standards for antibiotics in the environment; (2) further toxicological studies must be performed on potential chronic effects of antibiotic mixtures and their degradation products at environment-related concentration; (3) understand better and deeper the toxicity of antibiotics toward non-target organisms by proteomic responses and gene expression study; (4) human health may be affected by the consumption of agricultural products polluted by antibiotics, more attention should be paid to the indirect effects of antibiotics caused by the drinking water and food chain; (5) further research is needed to evaluate the relationship between antibiotic contamination and the presence of ARGs/ARB in the environment; (6) elucidation of degradation products and their bioactivity needs to be further investigated; (7) develop efficient and low-cost treatment technology for controlling the emission of antibiotic contaminants.

## Supplementary Material

Supplemental Material
