# Supplementary material for: Antibiotics: An overview on the environmental occurrence, toxicity, degradation, and removal methods
Source: Bioengineered. 2021 Oct 6;12(1):7376–416. doi: 10.1080/21655979.2021.1974657 (PMC8806427; doi:10.1080/21655979.2021.1974657)
Supplement: Supplemental Material [file KBIE_A_1974657_SM9494.doc]

***Supporting Information for***

**Antibiotics: An overview on the environmental occurrence, toxicity, degradation, and removal methods**

Qiulian Yang^1†^, Yuan Gao^1†^, Jian Ke^1^, Pau Loke Show^2^, Yuhui Ge^1^, Yanhua Liu^1^, Ruixin Guo^1^*, Jianqiu Chen^1^*

1. School of Engineering, China Pharmaceutical University, Nanjing 211198, China.

2. The University of Nottingham Malaysia, Jalan Broga, 43500 Semenyih, Selangor Darul Ehsan, Malaysia.

Corresponding author:

***Ruixin Guo & Jianqiu Chen**: School of Engineering, China Pharmaceutical University, 210009, Nanjing, China, Tel.: + 86 25 86185190, Fax: + 86 25 86185190. E-mail address: ruixinguo@cpu.edu.cn (R.X. Guo) & [cjqer@cpu.edu.cn](mailto:cjqer@cpu.edu.cn) (J.Q. Chen).

**^†^** Qiulian Yang & Yuan Gao contributed equally to this work.

**Table S1****. Physico-chemical properties of major antibiotics described in this review**

| Antibiotic | Abbreviation | CAS number | Molecular formula | Molecular weight (g/mol) | Water solubility (mg/L) | Log K_ow_/  Log P | pK_a_ | Chemical structure |
| --- | --- | --- | --- | --- | --- | --- | --- | --- |
| ***β-lactams*** |  |  |  |  |  |  |  |  |
| Amoxicillin | AMX | 26787-78-0 | C_16_H_19_N_3_O_5_S | 365.4 | 3,430^a^ | 0.87^a^ | 2.4^f^ | 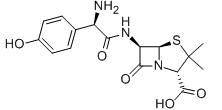 |
| Ampicillin | AMP | 69-53-4 | C_16_H_19_N_3_O_4_S | 349.4 | 10,100 (21°C)^a^ | 1.35^a^ | 1.45^f^ | 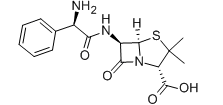 |
| Cephalexin |  | 15686-71-2 | C_16_H_17_N_3_O_4_S | 347.39 | 1789^a^ | 0.65^a^ | 2.56/6.88^g^ | 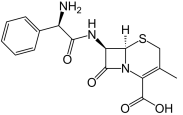 |
| Cefoxitin | CFX | 35607-66-0 | C_16_H_17_N_3_O_7_S_2_ | 427.45 | 105^b^ | -0.02^a^ | 3.59^*^ | 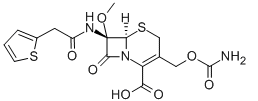 |
| Cephalothin |  | 153-61-7 | C_16_H_16_N_2_O_6_S_2_ | 396.44 | 158^a^ | 0^a^ | 3.63^*^ | 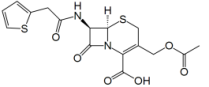 |
| Cephradine |  | 38821-53-3 | C_16_H_19_N_3_O_4_S | 349.4 | 21,300^b^ | 0.410^b^ | 2.63/7.30^g^ | 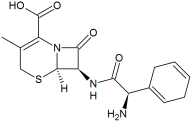 |
| Cephapirin |  | 21593-23-7 | C_17_H_17_N_3_O_6_S_2_ | 423.46 | 1030^a^ | -1.15^a^ | 2.15^a^ | 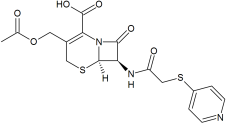 |
| ***Macrolides*** |  |  |  |  |  |  |  |  |
| Erythromycin | ERY | 114-07-8 | C_37_H_67_NO_13_ | 733.93 | 2,000 (28°C)^a^ | 3.06^a^ | 8.88 (25°C)^a^ | 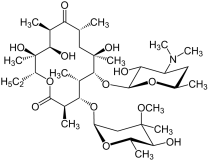 |
| Roxithromycin | RTM | 80214-83-1 | C_41_H_76_N_2_O_15_ | 837.05 | 0.0189 (25°C)^a^ | 1.7^a^ | 8.8^f^ | 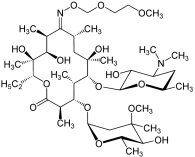 |
| Tylosin | TLS | 1401-69-0 | C_46_H_77_NO_17_ | 916.10 | 5^b^ | 1.63^b^ | 7.73^b^ | 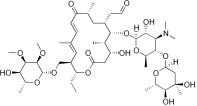 |
| Azithromycin | AZM | 83905-01-5 | C_38_H_72_N_2_O_12_ | 748.98 | 7.09^b^ | 4.02^a^ | 8.74 (25°C)^a^ | 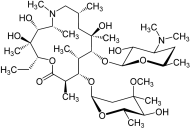 |
| Clarithromycin | CTM | 81103-11-9 | C_38_H_69_NO_13_ | 747.95 | 0.33^a^ | 3.16^a^ | 8.99 (25°C)^a^ | 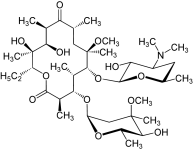 |
| Spiramycin | SPI | 8025-81-8 | C_43_H_74_N_2_O_14_ | 843.1 |  | 1.87^e^ | 8.0^e^ | 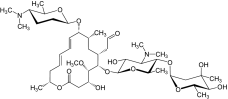 |
| ***Fluoroquinolones (FQs)*** | |  |  |  |  |  |  |  |
| Ciprofloxacin | CIP | 85721-33-1 | C_17_H_18_FN_3_O_3_ | 331.34 | 30,000 (20°C)^a^ | 0.28^a^ | 6.09^a^ | 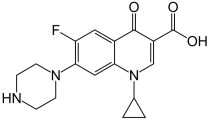 |
| Ofloxacin | OFL | 82419-36-1 | C_18_H_20_FN_3_O_4_ | 361.37 | 28,300^a^ | -0.39^a^ | 5.97/7.65 ^h^ | 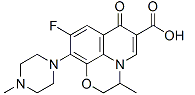 |
| Levofloxacin |  | 100986-85-4 | C_18_H_20_FN_3_O_4_ | 361.37 | Insoluble ^a^ | 2.1^a^ | 5.45/6.2^a^ |  |
| Norfloxacin | NOR | 70458-96-7 | C_16_H_18_FN_3_O_3_ | 319.33 | 178,000^a^ | -1.03^a^ | 3.11/6.10/ 8.6/10.56 ^h^ | 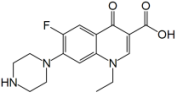 |
| Enrofloxacin | ENR | 93106-60-6 | C_19_H_22_FN_3_O_3_ | 359.4 | 3,400^b^ | 0.70^b^ | 3.85/6.19/7.59/9.86 ^d^ | 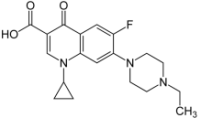 |
| Enoxacin | ENO | 74011-58-8 | C_15_H_17_FN_4_O_3_ | 320.32 | 34,300^b^ | -0.2^b^ | 6.04^h^ | 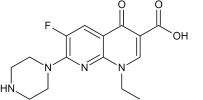 |
| Lomefloxacin | LOM | 98079-51-7 | C_17_H_19_F_2_N_3_O_3_ | 351.35 | 27,200^a^ | -0.3^a^ | 5.64/8.7^*^ |  |
| Moxifloxacin | MOX | 151096-09-2 | C_21_H_24_FN_3_O_4_ | 401.43 | 168^*^ | 2.9^a^ | 5.69/9.42^*^ | 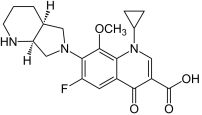 |
| ***Tetracyclines (TCs)*** |  |  |  |  |  |  |  |  |
| Tetracycline | TC | 60-54-8 | C_22_H_24_N_2_O_8_ | 444.44 | 231 (25°C)^a^ | -1.30^a^ | 3.3 (25°C)^a^ | 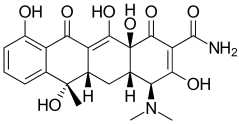 |
| Chlortetracycline | CTC | 57-62-5 | C_22_H_23_ClN_2_O_8_ | 478.88 | 630^b^ | -0.62^b^ | 3.3/7.55/ 9.33^d^ | 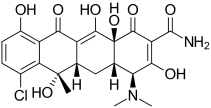 |
| Oxytetracycline | OTC | 79-57-2 | C_22_H_24_N_2_O_9_ | 460.43 | 313 (25°C)^a^ | -0.9^a^ | 3.27/9.5^d^ | 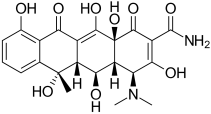 |
| Doxycycline | DXC | 564-25-0 | C_22_H_24_N_2_O_8_ | 444.4 | 630 (25°C)^a^ | -0.02^a^ | 7.75^*^ | 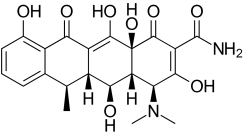 |
| Minocycline |  | 10118-90-8 | C_23_H_27_N_3_O_7_ | 457.476 | 52,000 (25°C)^a^ | 0.05^a^ | 8.25^*^ | 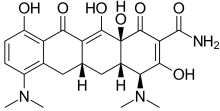 |
| ***Sulfonamides (SAs)*** | |  |  |  |  |  |  |  |
| Sulfamethoxazole | SMX | 723-46-6 | C_10_H_11_N_3_O_3_S | 253.28 | 610 (37°C)^a^ | 0.89^a^ | 1.97/6.16^*^ | 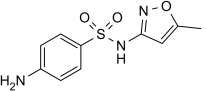 |
| Sulfadiazine | SDZ | 68-35-9 | C_10_H_10_N_4_O_2_S | 250.28 | 77 (25°C)^a^ | -0.09^a^ | 6.36^a^ | 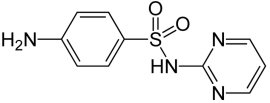 |
| Sulfamethazine | SMZ | 57-68-1 | C_12_H_14_N_4_O_2_S | 278.33 | 1500 (29ºC)^a^ | 0.89^a^ | 7.59^a^ | 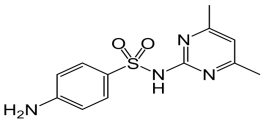 |
| Sulfathiazole | ST | 72-14-0 | C_9_H_9_N_3_O_2_S_2_ | 255.32 | 373 (25°C)^a^ | 0.05^a^ | 7.2^a^ | 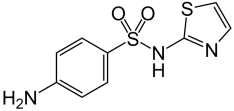 |
| Sulfapyridine | SPD | 144-83-2 | C_11_H_11_N_3_O_2_S | 249.29 | 268(25°C)^a^ | 0.35^a^ | 8.43^a^ | 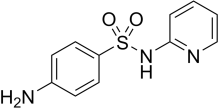 |
| Sulfamerazine | SM | 127-79-7 | C_11_H_12_N_4_O_2_S | 264.3 | 202 (20 °C)^a^ | 0.14^a^ | 2.01/6.99^*^ | 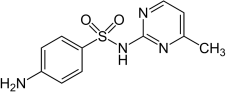 |
| Sulfamonomethoxine | SMM | 1220-83-3 | C_11_H_12_N_4_O_3_S | 280.31 | 4030 (25°C)^b^ | 0.7^b^ |  |  |
| Sulfamethoxypyridazine | SMP | 80-35-3 | C_11_H_12_N_4_O_3_S | 280.31 | 147 (37°C)^b^ | 0.32^b^ | 6.7^b^ | 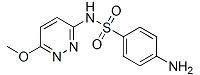 |
| Sulfanilamide |  | 63-74-1 | C_6_H_8_N_2_O_2_S | 172.2 | 7500 (25°C)^a^ | -0.62^a^ | 10.6 (20°C)^a^ | 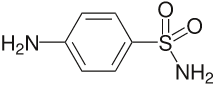 |
| ***Diaminopyrimidines*** | |  |  |  |  |  |  |  |
| Trimethoprim | TMP | 738-70-5 | C_14_H_18_N_4_O_3_ | 290.32 | 400 (25 ºC)^a^ | 0.91^a^ | 7.12^a^ | 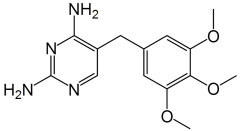 |
| ***Other classes*** |  |  |  |  |  |  |  |  |
| Vancomycin | VCM | 1404-90-6 | C_66_H_75_Cl_2_N_9_O_24_ | 1449.254 | 225^*^ | -3.1^a^ | 2.99/9.93^*^ | 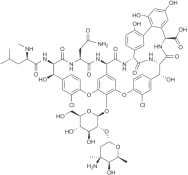 |
| Lincomycin | LIN | 154-21-2 | C_18_H_34_N_2_O_6_S | 406.54 | 927 (25ºC)^b^ | 0.56^a^ | 3.24/8.41^*^ | 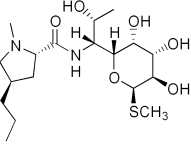 |
| Clindamycin | CLI | 18323-44-9 | C_18_H_33_ClN_2_O_5_S | 424.98 | 30.6^a^ | 2.16^a^ | 12.16/7.55^*^ |  |
| Chloramphenicol | CAP | 56-75-7 | C_11_H_12_Cl_2_N_2_O_5_ | 323.13 | 2500 (25°C)^a^ | 1.14^a^ | 5.5^e^ | 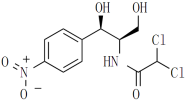 |
| Florfenicol | FF | 73231-34-2 | C_12_H_14_Cl_2_FNO_4_S | 358.2 | 5900^e^ | -0.12^e^ | 9.0^e^ | 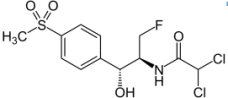 |
| ***Metabolite*** |  |  |  |  |  |  |  |  |
| N^4^-Acetyl- sulfadiazine |  | 127-74-2 | C_12_H_12_N_4_O_3_S | 292.3178 | 159 (37°C)^b^ | 0.390^b^ |  | 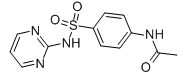 |
| N^4^-Acetyl- sulfamerazine |  | 127-73-1 | C_13_H_14_N_4_O_3_S | 306.345 | 280 (37°C)^b^ | 0.930^b^ |  |  |
| 14-(R)-hydroxy- clarithromycin ^c^ |  | 110671-78-8 |  |  |  |  |  |  |
| N-desmethyl- clarithromycin ^i^ |  | 101666-68-6 | C_37_H_67_NO_13_ | 733.93 |  |  |  | 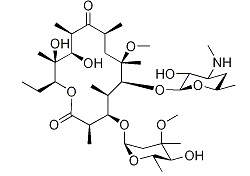 |

^a^ DRUGBANK, https://www.drugbank.ca/drugs; ^b^ U.S. National Library of Medicine ChemIDPlus Advanced. (https://chem.nlm.nih.gov/chemidplus/)

^c^ 1; ^d^ 2; ^e^ 3; ^f^ 4; ^g^ 5; ^h^ 6; ^i^ Chemical Book, http://www.chemicalbook.com/ProductChemicalPropertiesCB21176571_EN.htm [accessed 15 July 2021]

^*^ indicates predicted data. From DRUGBANK, https://www.drugbank.ca/drugs.

**References**

1. Baumann M, Weiss K, Maletzki D, Schussler W, Schudoma D, Kopf W, et al. Aquatic toxicity of the macrolide antibioticclarithromycin and its metabolites. Chemosphere 2015; 120:192-198.

2. Chen K, Zhou JL. Occurrence and behavior of antibiotics in water and sediments from the Huangpu River, Shanghai, China. Chemosphere 2014; 95:604-612.

3. Mitchell SM, Ullman JL, Teel AL, Watts RJ. Hydrolysis of amphenicol and macrolide antibiotics: Chloramphenicol, florfenicol, spiramycin, and tylosin. Chemosphere 2015; 134:504-511.

4. Papageorgiou M, Kosma C, Lambropoulou D. Seasonal occurrence, removal, mass loading and environmental risk assessment of 55 pharmaceuticals and personal care products in a municipal wastewater treatment plant in Central Greece. Sci Total Environ 2016; 543:547-569.

5. Wang XH, Lin AY. Phototransformation of cephalosporin antibiotics in an aqueous environment results in higher toxicity. Environ Sci Technol 2012; 46:12417-12426.

6. Zhang R, Zhang G, Zheng Q, Tang J, Chen Y, Xu W, et al. Occurrence and risks of antibiotics in the Laizhou Bay, China: impacts of river discharge. Ecotoxicol Environ Saf 2012; 80:208-215.

**Abbreviations**

**MAs:** macrolides; **FQs:** fluoroquinolones; **TCs:** tetracyclines; **SAs:** sulfonamides; **WWTPs:** wastewater treatment plants;

**SPE:** solid-phase extraction; **UAE:** ultrasound-assisted extraction; **QuEChERS:** quick, easy, cheap, effective, rugged, and safe;

**LC:** liquid chromatography; **MS/MS:** tandem mass spectrometry; **AMX:** amoxicillin; **CIP:** ciprofloxacin; **TMP:** trimethoprim;

**NOR:** norfloxacin; **SMX:** sulfamethoxazole; **DOX:** doxycycline; **LOQ:** limit of quantification; **SDGs:** sustainable development goals;

**ARGs:** antibiotic resistance genes; **TPs:** transformation products; **ND:** not detected; **ERY:** erythromycin; **OFL:** ofloxacin;

**CTC:** chlortetracycline; **OTC:** oxytetracycline; **4-ETC:** 4-epitetracycline; **4-EATC:** 4-epianhydrotetracycline;

**ERY-H_2_O:** anhydro-erythromycin; **RTM:** roxithromycin; **CTM:** clarithromycin; **SDZ:** sulfadiazine; **SMZ:** sulfamethazine;

**CAPs**: chloramphenicols; **DM:** dry matter; ***P. subcapitata*:** *Pseudokirchneriella subcapitata*; **SOD:** superoxide dismutase;

**MDA:** malondialdehyde; **CAT:** catalase; **GR:** glutathione reductase; **GSH:** glutathione; **ROS:** reactive oxygen species;

**DKAs:** β-diketone antibiotics; **AMA:** amoxicilloic acid; **ARB:** antibiotic-resistant bacteria; ***bla_TEM_:*** β-lactams resistance gene;

***erm*:** macrolide resistance gene; ***qnrS*:** fluoroquinolone resistance gene; ***tet*:** tetracycline resistance gene;

***sul*:** sulphonamide resistance genes; **qPCR** assays: Real-time PCR assays; **HSs:** humic substances; **HA:** humic acid
